# The genomic features of parasitism, Polyembryony and immune evasion in the endoparasitic wasp *Macrocentrus cingulum*

**DOI:** 10.1186/s12864-018-4783-x

**Published:** 2018-05-30

**Authors:** Chuanlin Yin, Meizhen Li, Jian Hu, Kun Lang, Qiming Chen, Jinding Liu, Dianhao Guo, Kang He, Yipei Dong, Jiapeng Luo, Zhenkun Song, James R. Walters, Wenqing Zhang, Fei Li, Xuexin Chen

**Affiliations:** 1 Ministry of Agriculture Key Lab of Molecular Biology of Crop Pathogens and Insects, Institute of Insect Science, Zhejiang University, 866 Yuhangtang Road, Hangzhou, 310058 China; 2 State Key Laboratory of Biocontrol, Sun Yat-sen University, 135 Xingang Road West, Guangzhou, 510275 China; 3 College of Information Science and Technology, Nanjing Agricultural University, Nanjing, 210095 China; 4 College of Plant Protection, Nanjing Agricultural University, Nanjing, 210095 China; 5 Ecology and Evolutionary Biology, University of Kansas, Lawrence, KS 66046 USA

**Keywords:** *Macrocentrus cingulum*, Genome, Polyembryony, Immune evasion, Comparative genomics

## Abstract

**Background:**

Parasitoid wasps are well-known natural enemies of major agricultural pests and arthropod borne diseases. The parasitoid wasp *Macrocentrus cingulum* (Hymenoptera: Braconidae) has been widely used to control the notorious insect pests *Ostrinia furnacalis* (Asian Corn Borer) and *O. nubilalis* (European corn borer). One striking phenomenon exhibited by *M. cingulum* is polyembryony, the formation of multiple genetically identical offspring from a single zygote. Moreover, *M. cingulum* employs a passive parasitic strategy by preventing the host’s immune system from recognizing the embryo as a foreign body. Thus, the embryos evade the host’s immune system and are not encapsulated by host hemocytes. Unfortunately, the mechanism of both polyembryony and immune evasion remains largely unknown.

**Results:**

We report the genome of the parasitoid wasp *M. cingulum*. Comparative genomics analysis of *M. cingulum* and other 11 insects were conducted, finding some gene families with apparent expansion or contraction which might be linked to the parasitic behaviors or polyembryony of *M. cingulum*. Moreover, we present the evidence that the microRNA miR-14b regulates the polyembryonic development of *M. cingulum* by targeting the c-Myc Promoter-binding Protein 1 (*MBP-1*), histone-lysine N-methyltransferase 2E (*KMT2E*) and segmentation protein *Runt*. In addition, Hemomucin, an O-glycosylated transmembrane protein, protects the endoparasitoid wasp larvae from being encapsulated by host hemocytes. Motif and domain analysis showed that only the hemomucin in two endoparasitoids, *M. cingulum* and *Venturia canescens,* possessing the ability of passive immune evasion has intact mucin domain and similar O-glycosylation patterns, indicating that the hemomucin is a key factor modulating the immune evasion.

**Conclusions:**

The microRNA miR-14b participates in the regulation of polyembryonic development, and the O-glycosylation of the mucin domain in the hemomucin confers the passive immune evasion in this wasp. These key findings provide new insights into the polyembryony and immune evasion.

**Electronic supplementary material:**

The online version of this article (10.1186/s12864-018-4783-x) contains supplementary material, which is available to authorized users.

## Background

Parasitoid wasps are a group of hymenopteran insects that parasitize the eggs, larvae or pupae of other arthropods [1]. These wasps differ from other parasitic organisms because they kill their host, and the adult wasps are free-living. Because of their host specificity and lethality, parasitoid wasps provide an important and effective strategy for the biological control of agricultural pests, thus reducing the need for chemical pesticides [2, 3]. Additionally, short generation times, ease of rearing, and interfertile species are characteristics that make at least some parasitoid wasps highly tractable genetic model systems [4]. This is exemplified by *Nasonia vitripennis*, which was the first parasitoid wasp genome to be sequenced, laying the foundation for genomic research in this economically and ecologically significant group of insects [5].

One striking phenomenon exhibited by numerous parasitoid wasp species is polyembryony, the formation of multiple genetically identical offspring from a single zygote. Polyembryony appears in only some parasitic species within four families of Hymenoptera and one species of Strepsiptera in insects, and is believed to have evolved independently at least four times among parasitoid wasps [6]. This includes the endoparasitic wasp *Macrocentrus cingulum,* for which some details of polyembryonic development have been described [6, 7]. *M. cingulum* usually deposits egg(s) into the larval hemocoel of corn borer, *Ostrinia furnacalis* or *O. nubilalis*. Subsequently, the egg cleaves into several dozens of primary embryonic cells, one of which may further develop into a morula containing dozens of secondary embryonic embryos (SEE). SEE may develop into an embryo, which developed into a larva, or a pseudogerm, which was finally consumed by hatched larvae [8]. Proliferation of embryos are mainly related to the egg cleavage and the formation of morula. Notably, these meticulous physical observations of polyembryonic development have not yet been complemented with molecular analyses. Thus, there remains tremendous opportunities for investigating the molecular mechanisms underlying the developmental complexity of polyembryony.

**Fig. 1:**
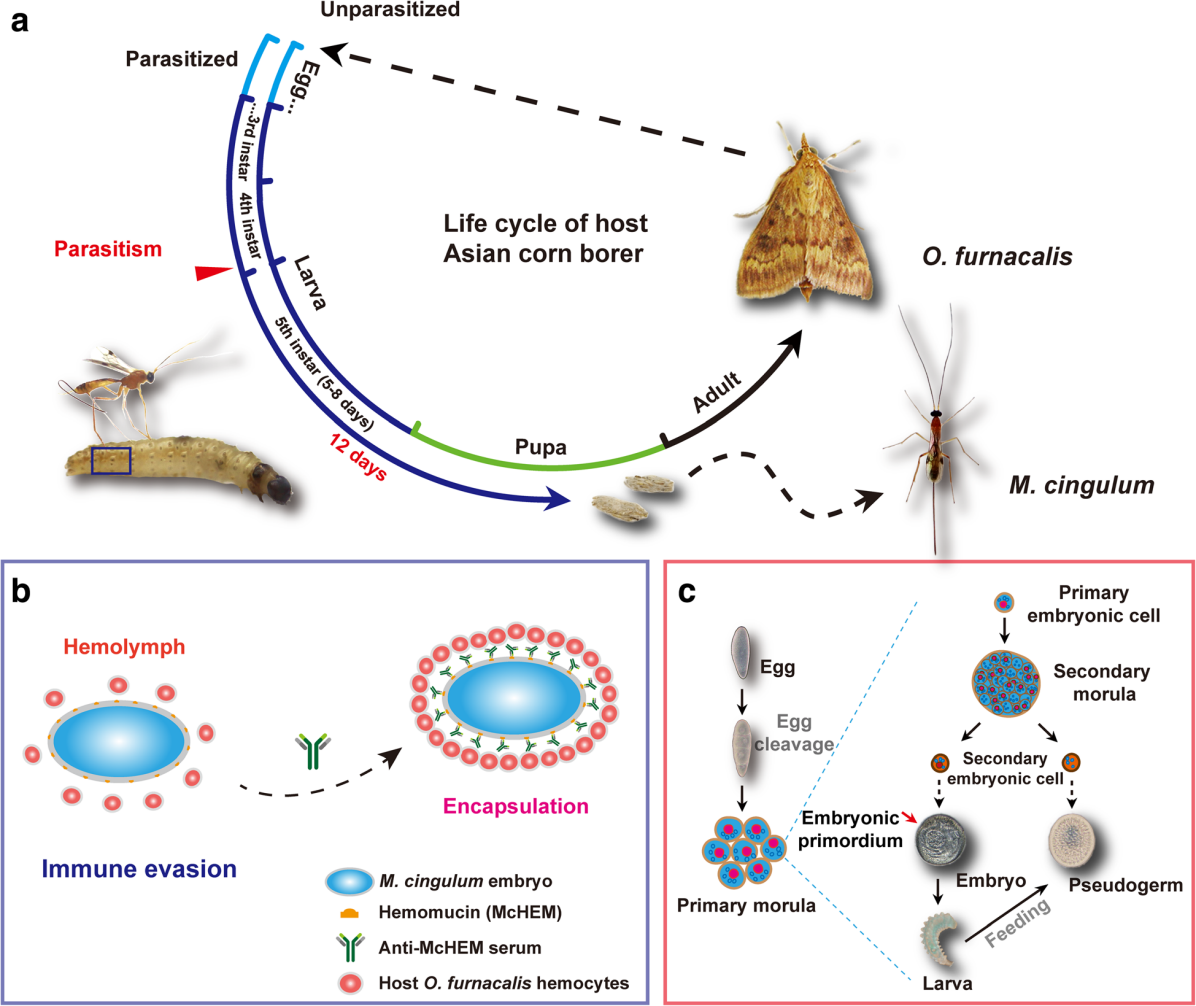
The life cycle and living strategies of *M. cingulum*. **a** The wasp *M. cingulum* parasitizes the larvae of Asian corn borer *Ostrinia furnacalis* at late stage of the 4th instar*.* The wasp develops in host hemocoel and grows into adult 12 days later. After parasitism, the host expands their larval stage from 5 to 8 days to 12–13 days, and never pupates. Eventually, the wasp larvae creep out of the corpse of host and cocoon on it, which has been consumed by wasp larvae. **b** The wasp *M. cingulum* evades the encapsulation of host hemocytes by Hemomucin. The immune evasion of *M. cingulum* is related to the O-glycosylation of the transmembrane protein Hemomucin located on the surface of egg and embryos. **c** The polyembryonic development of *M. cingulum*. One egg of *M. cingulum* can proliferate into multiple genetically identical offspring. The wasp egg firstly cleaves into dozens of primary embryonic cells, which split within the extraembryonic membrane and grows into morulae containing dozens of secondary embryonic cells, which differentiated into two castes: embryo and pseudogerm. Embryo is composed of embryonic primordium and syncytial extraembryonic membrane, and eventually develops into wasp larva. Pseudogerm is syncytium and is eaten by the wasp larva at the late stage as compensatory nutrition

Beyond polyembryony, parasitoid wasps exhibit a range of other distinct and noteworthy traits that evolved as strategies to manipulate their host. For instance, many species introduce various parasitoid-associated factors such as venom and polydnaviruses (PDV) into the host during oviposition to block the host immune responses [9, 10]. Another example is the production of teratocytes from cellular membranes after the eggs hatched. The teratocytes are released into the hemolymph of the host to inhibit melanization and to produce anti-microbial peptides to protect the host from being infected by other organisms [11]. However, in seeming contrast with these distinctly antagonistic parasitic tactics, *M. cingulum* employs a more passive parasitic strategy (Fig. 1) [12]. Parasitism substantially retards development of the *Ostrinia* host, ultimately allowing the adult wasps to emerge and kill the host before it pupates (Fig. 1a). While growing in the host hemolymph, *M. cingulum* embryos express surface features that prevent the host’s immune system from recognizing the embryo as a foreign body, thus the embryos evade the host’s immune system and are not encapsulated by host hemocytes (Fig. 1b) [12]. Available evidence suggests Hemomucin, an O-glycosylated transmembrane protein on the egg and embryo’s surface of *M. cingulum*, may protect the endoparasitoid wasp from being encapsulated by host hemocytes [13, 14]. When the expression of hemomucin was decreased by RNAi, more embryos were encapsulated relative to the control [13]. Similar results were observed after the embryos were digested by O-glycosidase, which may specifically digest β-gal (1 → 3) linkages between GalNAc and Ser/Thr of the mucin domain in hemomucin, indicating the important role of the mucin domain in hemomucin [13]. Despite these initial insights, the mechanism of immune evasion remains largely unknown.

With the aim of further investigations of polyembryony and immune evasion, along with other studies of wasp biology, we present the draft genome of *M. cingulum*. Comparative genomic analyses indicated that parasitic behavior has shaped the endoparasitoid wasp genome and induced significant gene gain and loss associated with parasitism. RNA-Seq analysis of the primary and secondary embryonic cells revealed that microRNA (miRNA) miR-14b was abundant in the primary embryonic cell. Target prediction and dual-luciferase assay validation suggested that miR-14b plays critical roles in polyembryonic development. Comparative analyses of wasp hemomucin genes found that the presence of a mucin domain and its glycosylation was positively associated with immune evasion in parasitoid wasps.

## Results

### Genome sequencing, assembly and annotation

We sequenced the genome of *M. cingulum* from ~ 1000 male wasps of an inbred strain which was maintained by sibling mating for five generations. Three paired-end libraries with different insert sizes (170 bp, 500 bp, 800 bp) and one mate-pair libraries with insert size 8 Kb (Additional file 1: Table S1), were constructed and sequenced using Illumina HiSeq 2000 platform, yielding 103.67 Gb of raw data. After removing the low-quality reads, 93.24 Gb of clean data were obtained, covering ~ 690 X of *M. cingulum* genome which was estimated to be 135 Mb by 17 K-mer analysis (Additional file 1: Figure S1, Additional file 1: Table S2) and flow cytometry (Additional file 1: Figure S2). The heterozygosity rate of the genome was estimated to be 0.2%, which was consistent with previous reports from other wasps and showed that Hymenoptera species generally have low heterozygosity [15].

We used ABySS Ver2.0.2 [16] to de novo assemble the genome, achieving a draft genome of 132 Mb with GC content of 35.66% (Table 1, Additional file 1: Table S3). The Contig N50 of the genome assembly was 64.9 Kb, which is among the largest of all published insect genomes (Additional file 1: Figure S3). We evaluated the genome assembly using Benchmarking Universal Single-Copy Orthologs (BUSCO v3) [17, 18], which identified 99.5% of 1658 conserved arthropod genes, of which 98. 9% (1639) are full length, indicating that the genome assembly contained almost all gene information and was suitable for subsequent analyses.

**Table 1:** Feature of assembled genome and Gene Sets

Features	*Macrocentrus cingulum*	*Nasonia vitripennis*	*Ceratosolen solmsi*
Genome size (Mb)	132.36	295	278
Number of contigs	13,289	26,605	15,018
Number of Scaffolds	5696	6181	7397
Quality Control (Covered by assembly)			
Contig N50 (bp)	64,884	18,500	74,395
Scaffold N50 (Kb)	192.445	709.00	9558
BUSCO genes (%)	99.45	95.96	60.19
Genomic Features			
Repeat (%)	24.9	42.1	–
G + C (%)	35.66	40.6	–
Gene Annotation			
Number of Genes	11,993	18,882	11,412

We annotated 145,038 repeat sequences (29 Mb), constituting 21.9% of the *M. cingulum* genome. Only nine repeat sequence families were identified in *M. cingulum* by RepeatModeler [19]. To date, *M. cingulum* ranks the third smallest known insect genome assemblies, only larger than 108 Mb of the human body louse *Prediculus humanus humanus* [20] and 89.6 Mbp of the midge *Belgica antarctica* [21]. Corresponding to its small size, the *M. cingulum* genome only contains 15.6% known transposable elements and 1.8% tandem repeats. In contrast, another parasitic wasp *N. vitripennis* had a greater abundance of transposable elements and repetitive DNA (> 30%) [5]. *N. vitripennis* also has a larger genome (295 Mb) than *M. cingulum*, suggesting that the percentages of repeat elements is the main factor affecting the genome size (Additional file 1: Table S6).

We used the Optimized Maker-based Insect Genome Annotation (OMIGA) pipeline [22] to annotate protein coding genes in the *M. cingulum* genome. The OMIGA pipeline included training the de novo prediction software AUGUSTUS and SNAP with 5036 full-length *M. cingulum* genes that were obtained by analyzing the transcriptomes of embryos, larvae, adult male and female wasps. After integrating evidence from RNA-Seq, de novo prediction and homolog protein alignment, an official gene set (OGS) of 11,993 genes for *M. cingulum* was obtained. 98.6% of OGSs had the expression evidence in at least one sample. In total, 96.86% of the inferred proteins found the homology sequences in the databases of NCBI NR, SWISS-PROT and InterPro (Table 1 and Additional file 1: Table S7).

The average intron length in the *M. cingulum* genome was only 385 bp, much less than other hymenopteran insects such as 1285 bp in *N. vitripennis* and 1291 bp in *Apis mellifera.* The short intron length in *M. cingulum* was consistent with its small genome size. However, the average CDS length is similar among three hymenopterans, 1536 bp in *M. cingulum*, 1260 bp in *A. mellifera* and 1289 bp in *N. vitripennis*, suggesting that the CDS lengths were conserved whereas the intron lengths might be variable in closely related species (Additional file 1: Table S8).

We identified noncoding RNA by homologous search against the Rfam database [23] (E-value <=1e-5), yielding 16 small nucleolar RNA (snoRNA), 39 small nuclear RNA (snRNA), 144 transfer RNA (tRNA) and 148 ribosome RNA (rRNA). We predicted 111 miRNA using the software MapMi [24] with the Hexapoda miRNAs in the miRbase as the reference (Additional file 1: Table S7) [25].

### Genome-based phylogeny of *M. cingulum*

**Fig. 2:**
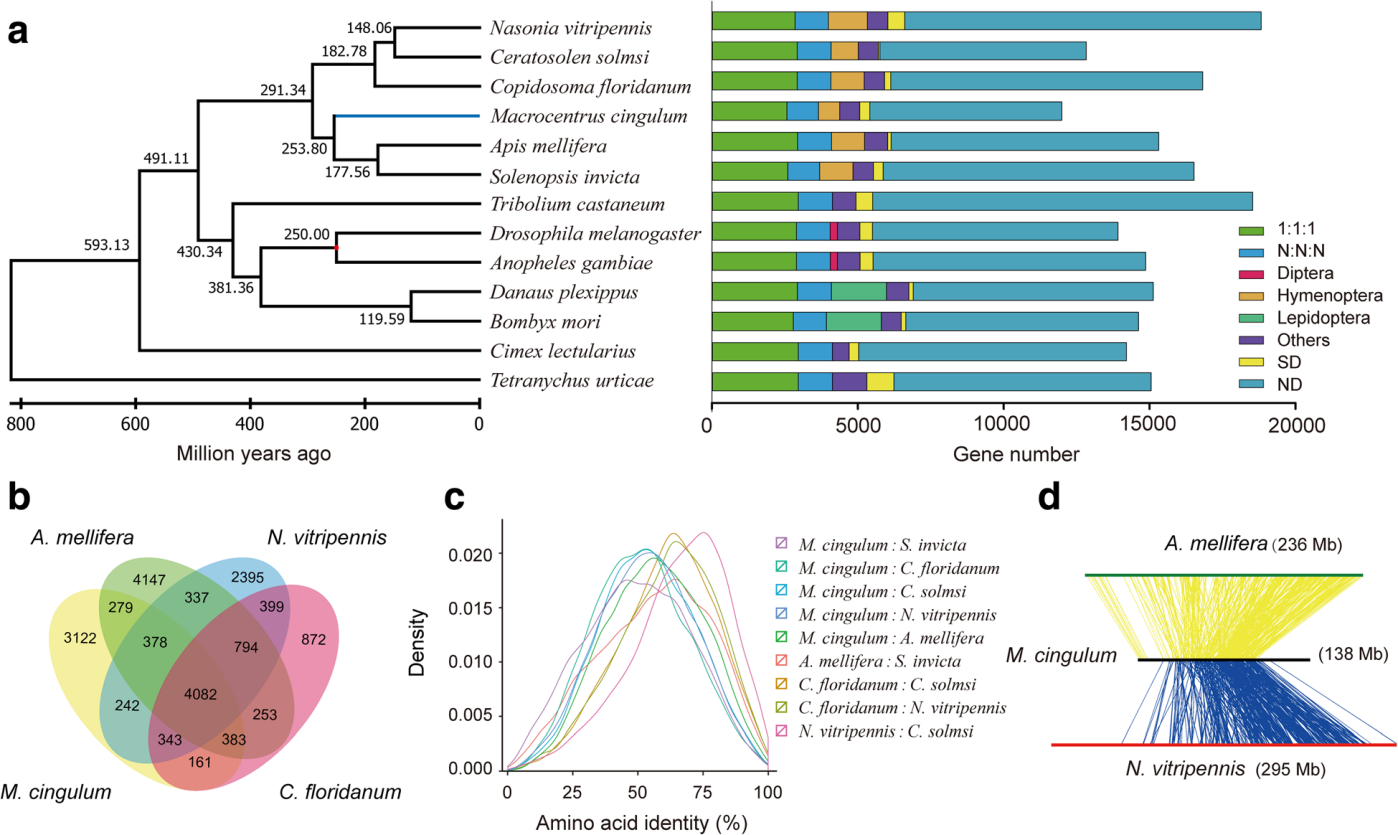
Phylogenetic and comparative genomics analysis of *M. cingulum* genome. **a** The phylogenetic tree was constructed from 532 single-copy genes with 47,606 reliable sites by RAxML maximum likelihood methods. Bars are subdivided to represent different types of orthologs’ relationships. 1:1:1 indicates universal single-copy genes, absence and/or duplication in a single genome was allowed. N: N: N indicates other universal genes, absence in a single genome or two genomes within the different orders was allowed. Diptera indicates dipteran-specific genes. Lepidoptera indicates lepidopteran-specific genes. Hymenoptera indicates hymenopteran-specific. Others indicates all other orthologs. SD, species-specific duplicated genes. ND, species-specific genes. **b** The Venn diagram indicates the numbers of orthologous genes shared among the four hymenopterans, *M. cingulum*, *C. floridanum*, *A. mellifera* and *N. vitripennis*. All four hymenoptera insects share 4082 common homologous genes. The data shows the completeness of the gene repertoire encoded in the wasp *M. cingulum* genome. **c** The distribution of pairwise amino acid identity between six hymenoptera insects. Histogram shows *M. cingulum* shares higher amino acid identity with *A. melifera* than with other hymenopterans. There were 6796 orthologs between *A. melifera* and *S. invicta*, 4452 orthologs between *C. floridanum* and *C. solmsi*, 5137 orthologs between *C. floridanum* and *N. vitripennis*, 6403 orthologs between *M. cingulum* and *A. melifera*, 4196 orthologs between *M. cingulum* and *C. floridanum*, 4872 orthologs between *M. cingulum* and *C. solmsi*, 5808 orthologs between *M. cingulum* and *N. vitripennis*, 5616 orthologs between *M. cingulum* and *S. invicta*, 5790 orthologs between *N. vitripennis* and *C. solmsi*. **d** Microsynteny between three wasps by tracking gene positions through multiple species. The *M. cingulum* and *A. mellifera* shared 7284 orthologous, but only 1950 genes constituted 351 synteny blocks. The *M. cingulum* and *N. vitripennis* shared 6809 orthologous genes, but only 1877 genes constituted 346 synteny blocks, suggesting the frequent chromosome rearrangement among the wasp species

We carried out orthologous and homologous group analysis of proteins from 13 species, including six hymenopterans, two dipterans, two lepidopterans, one coleopteran, one hemipteran, and one mite species, finding 2957 single-copy orthologs and 1174 multi-copies orthologs (Fig. 2a). The genome of the mite *Tetranychus urticae* was used as the outgroup. We also found 2028 hymenopteran-specific genes and 5206 insect-specific genes. We performed a phylogenomic analysis of 589 single copy orthologous genes with 128,070 conserved sites using maximum likelihood method as implemented in RAXML [26]. The phylogenetic tree of these 13 species showed that *M. cingulum* and the other 5 wasps/ants formed a hymenoptera cluster. Unexpectedly, *M. cingulum* had a close relation with bees/ants rather than with the ectoparasitic wasp *N. vitripennis* in the family of Pteromalidae*. A. mellifera* diverged from *Solenopsis invicta* approximately 177 million years ago, whereas *M. cingulum* separated from them approximately 253 million years ago (Fig. 2a). Homology analysis also indicated that *M. cingulum* shared 5122 homologous genes with *A. mellifera*, higher than 5045 with *N. vitripennis* and 4969 with *Copidosoma floridanum* (Fig. 2b). Moreover, *M. cingulum* shared higher amino acid identity with *A. mellifera* compared With *N. vitripennis* or *C. floridanum* (Fig. 2c). Only 26.8% of the orthologous pairs between *M. cingulum* and *A. mellifera* shared a consensus gene order, suggesting that frequent occurrence of chromosomal rearrangement after divergence (Fig. 2d). Taken all these evidences together, genome-based phylogenetic analysis showed that the *M. cingulum* clustered together with bees/ants, rather than other parasitoid wasps such as *N. vitripennis* and *C. floridanum*, which is consistent with a previous Bayesian phylogenetic analysis with a conserved gene family [27]. We noticed that this result is not consistent with a recent report on the evolution of the Hymenoptera [28]. It remains possible that the sample bias in our work resulted in this inconsistence since we did not choose much more hymenopteran species. However, both genome-based phylogeny and homology analysis indicated that *M. cingulum* is closer to bees/ants rather than parasitoid wasps. This inconsistency is worthy of further clarification.

### The genomic features associated with parasitism

**Fig. 3:**
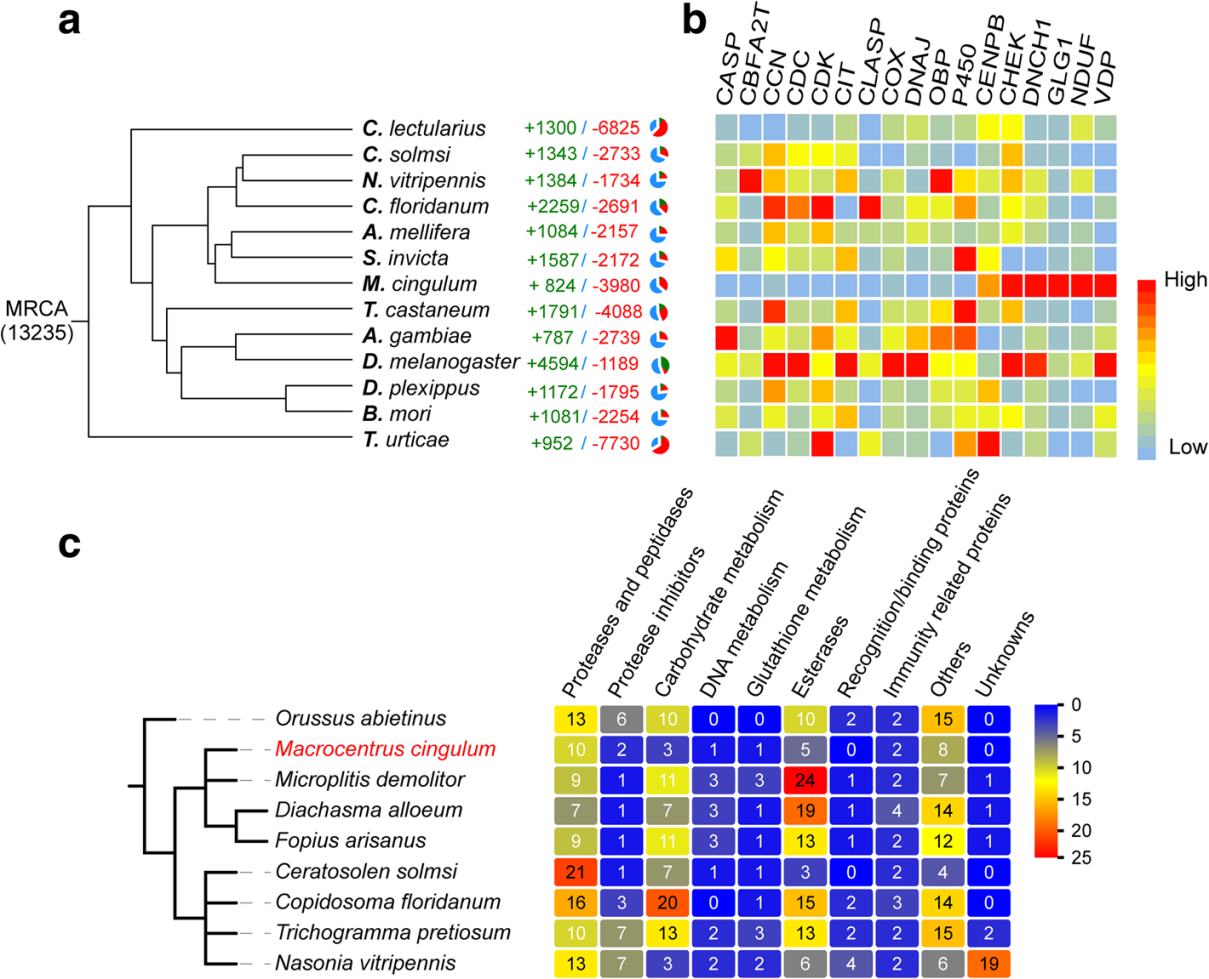
Gene gain and loss associated with parasitism. **a** Number of gene families with apparent expansion/contraction among *M. cingulum* and 12 other species. *M. cingulum* has a significant higher number of gene contractions than expansions. In contrast, *C. floridanum* has more events of gene expansions. **b** Gene families with significant contraction or expansion. Noticeably, the gene families of OBP, P450, CCN, CDC, CDK, CASP, CBFA2T, CIT, CLASP, COX, DNAJ were contracted, whereas the gene families of CHEK, DNCH1, DLG1, NDUF and VDP were expanded in *M. cingulum*. **c** Numbers of venom proteins in different parasitic wasps. *M. cingulum* has few venom proteins since it has the feature of immune evasion and does not rely on the venom proteins to condition the host

We identified gene families with apparent expansions or contractions in *M. cingulum* by a comparison with other five hymenopterans (including two parasitoid wasps), two dipterans, two lepidopterans, one coleopteran, and one hemipteran (Fig. 3). We observed some gene families were significantly contracted in *M. cingulum* and other parasitoid wasps (*p* < 0.05, student t-test), such as odorant binding proteins (OBP), P450 enzymes, Glutathione S-transferases (GST) (Additional file 1: Table S13 and S14, Additional file 1: Figure S8-S16). The contraction of OBP gene family could be related to the parasitoid lifestyle of *M. cingulum* because the parasitoid wasps obtain nutrients from the host. As such, it would be unnecessary for them to detect as many odorants as non-parasitic insects since they have a safer environment and abundant food sources (Additional file 1: Figure S12). It has been reported that OBP genes in another polyembryonic wasp *C. floridanum* showed caste-specific functions. The majority of OBP genes might have the functions in contacting with host hemolymph [29]. The decrease of P450 and GSTs gene families suggests the detoxification ability of the parasitoid wasp has degenerated, again perhaps because the host protects the parasitoid larvae from the external environment. The centromere protein and cyclin-dependent kinase have essential roles in cell cycle and cell division. These gene families were also significantly contracted in *M. cingulum* (*p* < 0.05, student t-test). The driving forces leading to the contraction of these gene families remain unclear. In addition, the venom proteins were significantly reduced in the *M. cingulum,* which is consistent with the fact that this endoparasitoid wasp does not rely on the venom proteins to control the host (Fig. 3c). Some insect immune genes are also contracted in *M. cingulum* (Additional file 1: Table S15). We found that the expansion of some gene families is associated with the parasitoid life. Several gene families that are critical in vesicular trafficking pathway were expanded, including ADP ribosylation factors (*ARF*) and cytoplasmic dynein. These expansions could facilitate the parasitoids to better absorb the nutrients from the host.

### The gene family expansions associated with polyembryony

*M. cingulum* has a striking characteristic of polyembryony. During the proliferation phase of the embryo development in *M. cingulum*, the blastomeres divide and generate dozens of secondary embryonic cells without differentiation [13]. Though the polyembryony is a striking reproductive strategy, its molecular mechanisms remain largely unknown. The gene family analysis also indicated *M. cingulum*’s genomic features associated with polyembryony. Euchromatic histone-lysine N-methyltransferase (*EHMT*) and Golgi apparatus protein (*GLG*) gene families are significantly expanded. *EHMT* have been implicated in both maintenance of pluripotency and stabilization of a differentiated cell identity [30, 31]. *GLG* interacts selectively and non-covalently with fibroblast growth factors that are key players in the processes of proliferation and differentiation of wide variety of cells and tissues [32]. Some orthologous groups specifically expanded in the two polyembryony wasps, such as vascular endothelial growth factor receptor which has been reported to control intestinal stem cell proliferation [33].

**Fig. 4:**
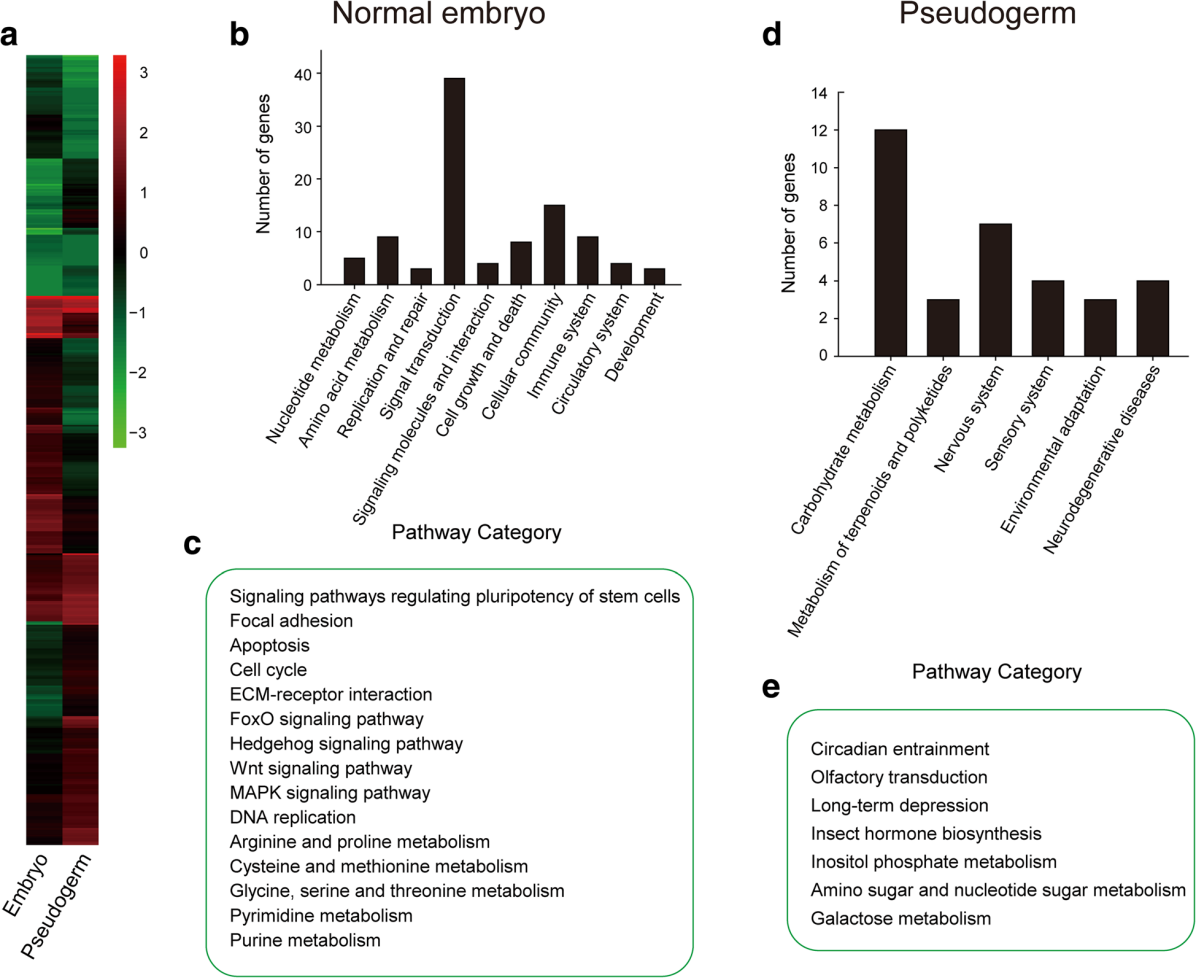
The differential gene expression analysis between embryos and pseudogerms of *M. cingulum*. **a** Heatmap of differentially expressed genes in embryos and pseudogerms. The signal transduction pathways were enriched in the embryos (**b**) whereas the pathways of carbohydrate metabolism were enriched in the pseudogerms (**d**). The enriched pathways were consistent with the fate and function of embryos (cell cycle, FOXO, Wnt, Hedgehog, etc.) (**c**) and pseudogerms (hormone biosynthesis, inositol phosphate metabolism, amino sugar and nucleotide sugar metabolism, etc.) (**e**)

Immediately upon oviposition, the *Macrocentrus* egg initiates cleavage and then enters the proliferation phase. During this stage, one single egg produces dozens of normal embryos and thousands of pseudogerms. The normal embryos produce embryonic primordia that develop into larvae while the pseudogerms do not. To identify the genes modulating polyembryonic development in *M. cingulum*, we performed RNA-Seq analysis of different types of embryos and compared the transcript abundances of two types of embryos (normal embryos and pseudogerms) (Fig. 4a). In normal embryos, the most abundant transcripts were from genes associated with cell growth and development, nucleotide and amino acid metabolism, DNA replication, purine and pyrimidine metabolism (Fig. 4b and c). In contrast, for the pseudogerms, a kind of abnormal embryo cells originated from the secondary embryonic cells and mainly provides protection and nutrients to the embryo, abundant transcripts were from genes associated with amino sugar and nucleotide sugar metabolism, galactose metabolism, inositol phosphate metabolism and insect hormone biosynthesis (Fig. 4d and e). The differences in highly-expressed genes between these two types of embryonic cells clearly reflects their distinct functions in embryonic development (Additional file 1: Table S11).

### The microRNA miR-14b modulates the polyembryony in *M. cingulum*

We also compared miRNA abundance between morulae, embryos, and pseudogerm. Pseudogerm is a syncytium, and it does not contain an embryonic primordium while the embryo does. The miR-14b stood out as having high-expression levels specifically in the primary morula, the early embryos at the proliferation phase, compared to normal embryos (*p* < 0.05, student t-test) (Additional file 1: Table S12). miR-14 is a conserved insect-specific miRNA. It was found to be an important regulator in insulin production and metabolism [34], hedgehog pathway [35], apoptosis [36], insect development and metamorphosis [37]. miR-14b shares identical seed region with miR-14 and has 3 different nucleotides near 3′ end compared with miR-14. Quantitative real time PCR (qPCR) further confirmed the high expression of miR-14b specifically in morulae but not in normal embryos (Additional file 1: Figure S17). We predicted the targets of miR-14b using five different software packages: miRanda, RNAhybrid, RNA22, PITA and TargetScan. Taking the intersection of results from all five packages, three genes were consistently predicted to be the target of miR-14b: c-Myc Promoter-binding Protein 1 (*MBP-1*), histone-lysine N-methyltransferase 2E (*KMT2E*) and segmentation protein *Runt*.

**Fig. 5:**
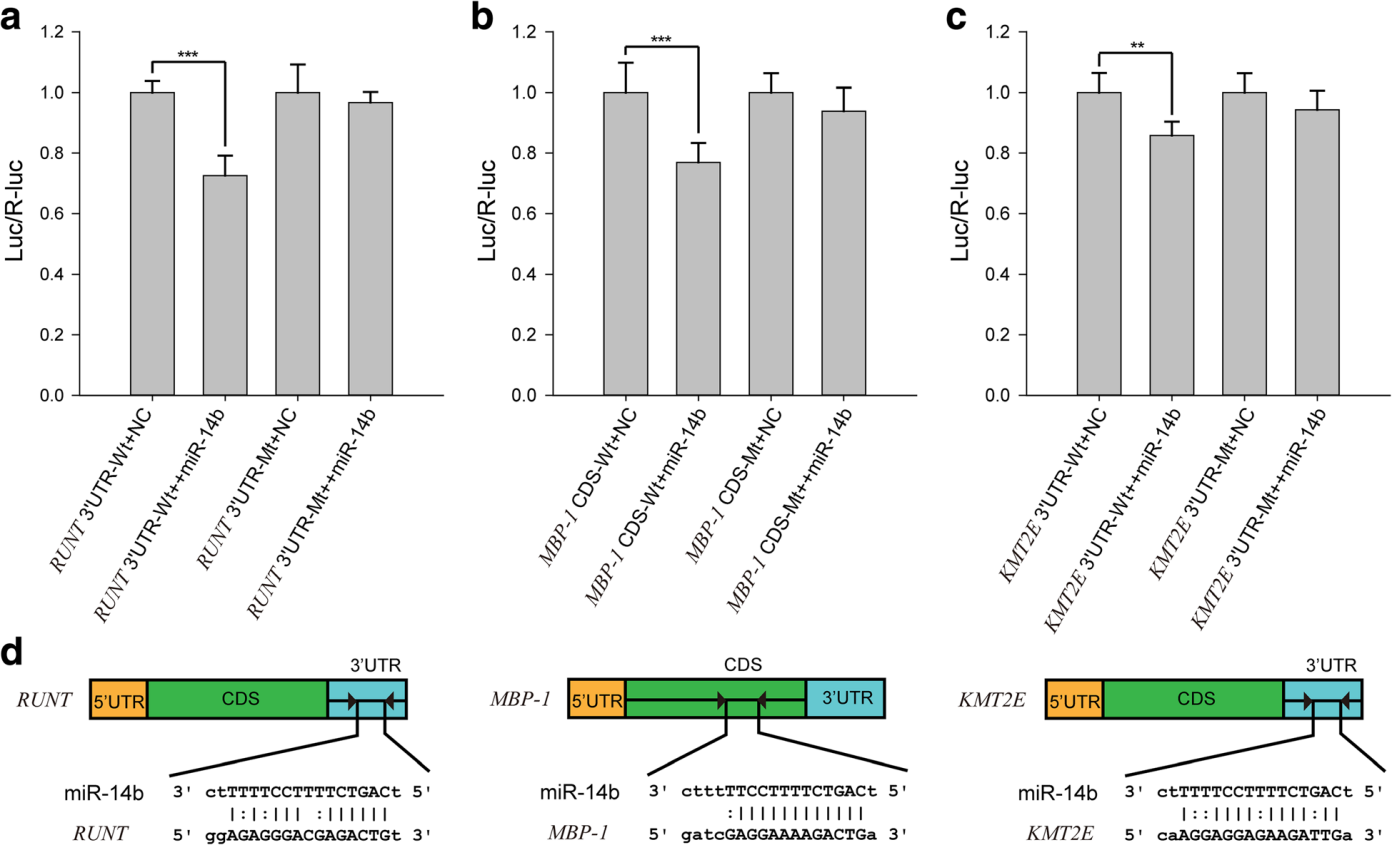
miR-14b modulate the polyembryony by targeting the *RUNT*, *MBP-1* and *KMT2E* in *M. cingulum*. **a**-**c** Dual-luciferase reporter assays were performed to examine the interaction of mci-miR-14b and its target sequences and corresponding mutated targeting sequences (RUNT 3’UTR-Mt, MBP-1 CDS-Mt, KMT2E 3’UTR-Mt) cloned into the 3’UTR of the reporter gene. Signals from the reporters with the 3’UTR of *RUNT*, *MBP-1*, *KMT2E* were significantly knocked down in the presence of the mci-miR-14b mimics (*p* < 0.0001). Introducing the mutations at the target sites abolish the silence effects. ** indicates 0.001 < *p* < 0.05, *** indicates *p* < 0.001. **d** The target sites of mci-miR-14b appears in the 3’UTR of *RUNT* and *KMT2E* whereas in the CDS of *MBP-1*

We performed a dual-luciferase assay to confirm the interactions between the miR-14b and three predicted target genes. The miR-14b binding regions on three target genes (3’UTRs of *KMT2E* and *Runt*, the CDS region of *MBP-1*) were introduced into the pMIR-REPORT vector at the downstream of a firefly luciferase gene. For the negative control, mutations were introduced into the target genes to abolish the miRNA target sites. The constructs that did not contain the miR-14b binding sites of the target genes were used as the negative control. All the constructs were transfected into HEK293T cells. Compared to the negative controls, the luciferase reporter activity of three positive constructs was significantly reduced in the presence of miR-14b mimics and the mutation of binding sites abolished the repressions (Fig. 5a, b, and c). These results confirmed that miR-14b targets on the *MBP-1*, *KMT2E* and *Runt*. The binding sites of the miRNA in the target genes are shown in Fig. 5d.

The first target gene *MBP-1* acts as a negative regulatory factor for the transcription factor *c-Myc*, which drives cell proliferation and regulates stem cell self-renewal [38]. miR-14b might control the *MBP-1* and thus stimulate the *c-Myc* to maintain the embryo cells to be pluripotent and to produce multiple normal embryos [39]. The second target gene *KMT2E* specifically mono- and di-methylates Lys-4 of histone H3 and thus regulates the expression of the downstream genes. *KMT2E* acts as an important cell cycle regulator, participating in cell cycle regulatory network [40]. The third target gene *Runt* is a vital transcriptional regulator which regulates the segmentation in the development. *Runt* plays an essential role in modulating the formation of classic developmental patterns [41]. Downregulation of *Runt* by miR-14b might prevent the transition from cell differentiation to cell proliferation phases of development.

### O-glycosylation of the mucin domain in hemomucin confers the immune evasion

**Fig. 6:**
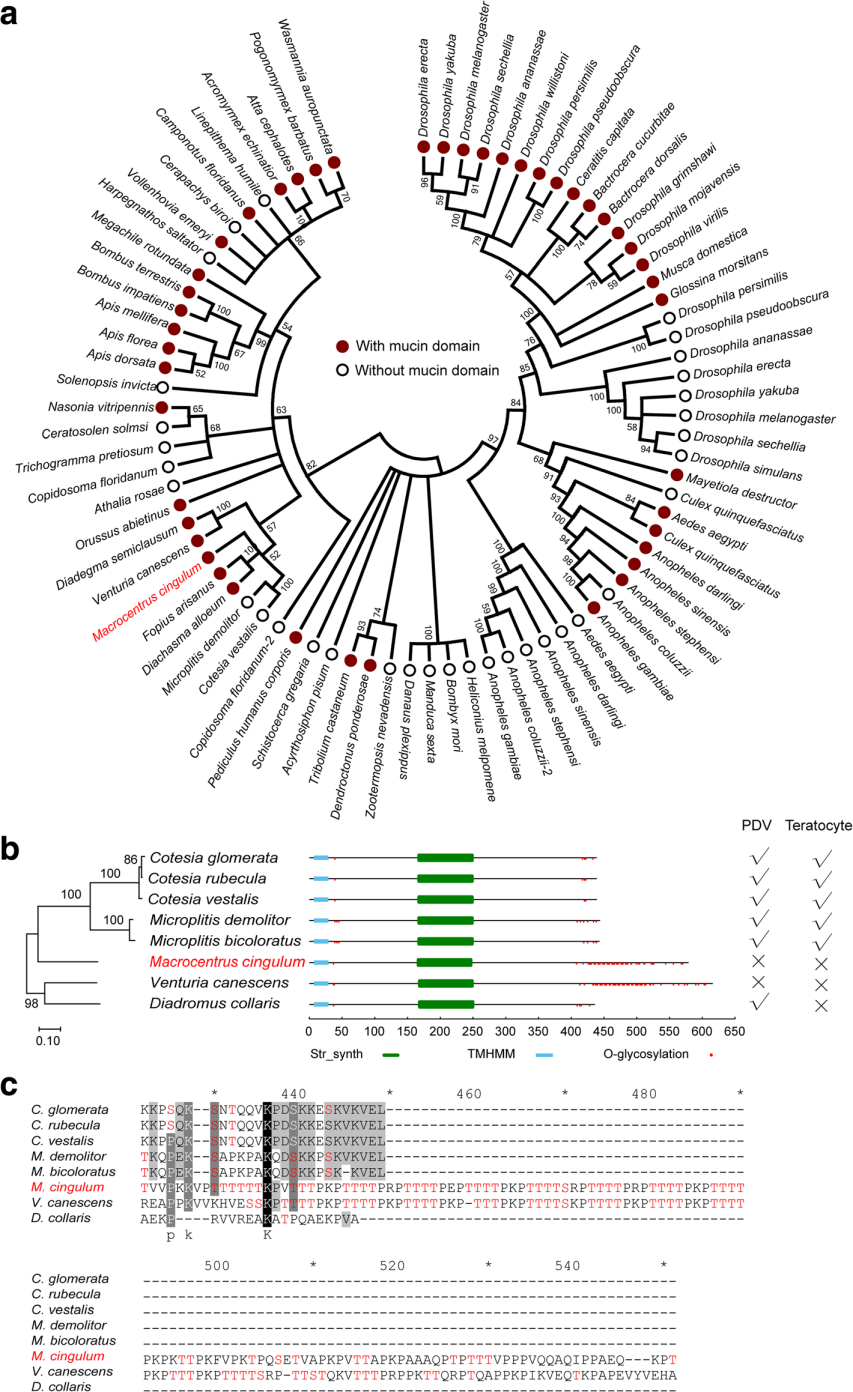
The phylogenetic relationships of insect hemomucin genes and the associations of mucin domain with immune evasion in *M. cingulum*. **a** the hemomucin genes of 69 insects were collected from the NCBI GenBank. Among which, 15 insects have two hemomucin genes, one with intact mucin domain and the other without mucin domain. Thirty-two insects have only one hemomucin gene with mucin domain whereas 22 insects have only one hemomucin gene without mucin domain. Phylogenetic analysis indicated that some wasps lost the mucin domain such as *Cotesia vestalis* and *Microplitis demolitor* whereas other wasps have mucin domain. **b** For the larval parasitoids, we found that only two wasps (*M. cingulum* and *V. canescens*) with immune evasion have the mucin domain whereas other wasps with PDV or teratocytes (or both) do not have. **c** The mucin domains have abundant O-glycosylation sites. The O-glycosylation status of the mucin domain involved in conferring the immune evasion in *M. cingulum* (unpublished data)

Most endoparasitoids actively inhibit the immune responses of the host by multiple parasitic factors such as PDV, teratocytes and venom. However, some endoparasitoid wasps, such as *M. cingulum,* successfully evade the encapsulation of host hemocytes by deploying *hemomucin*, an O-glycosylated protein. In *M. cingulum,* the hemomucin protein (McHEM) was shown to be highly expressed on the extraembryonic membrane of eggs and embryos. Either blocking with anti-McHEM serum or digestion with O-glycosidase led to the encapsulation of *M. cingulum* embryos by the host [13]. To better understand the genetic features of immune evasion, we searched the genomes or transcriptomes of eight endoparasitoid wasp species, and results showed that *hemomucin* exists in all wasps (Additional file 1: Table S10). Only *M. cingulum* and *Venturia canescens* hemomucin proteins contain a mucin domain with the typical stretches of poly-threonine separated by proline residues [42] (Fig. 6a). Both *M. cingulum* and *V. canescens* passively evade host cellular immune reactions [14, 43, 44]. In *V. canescens,* the hemomucin-lipophorin complex is thought to protect the parasitoid egg by camouflaging the egg surface with host proteins during the initial contact with hemolymph. In contrast, the other six larval parasitoid wasps we examined employ multiple parasitic factors such as PDV and teratocytes to actively suppress the immune reaction of host [45] (Fig. 6b). Prediction of O-glycosylation sites in the mucin domain showed that hemomucin of these two endoparasitoid wasps had similar O-glycosylation patterns (Fig. 6c). These pieces of evidence suggest that O-glycosylation in the mucin domain should be a key factor modulating the immune evasion in *M. cingulum*.

## Discussion

The comparative genomics analyses of the parasitoid wasp genomes revealed that the parasitoid lifestyle seems to have shaped the *M. cingulum* genome, including significant gene family contractions of OBP and metabolic detoxification enzymes (P450 and GST). Because the larvae residing in the host hemolymph are protected from the external complex environments, the contractions of OBP, P450 and GST are common features of parasitoid wasps [5, 46]. We also observed a decrease of the number of venom proteins in *M. cingulum*, which probably occurred because this endoparasitoid does not rely on its venom to condition the host. In contrast, the ARF and cytoplasmic dynein were significantly expanded, which may facilitate the transfer of nucleic acids, amino acids and other nutrients from the host hemolymph to the wasp larvae. We did not find any significant evidence for contraction or expansion of genes associated with circadian rhythm, immune system and digestion. Changes in these gene families might be expected because the endoparasitoid wasp larvae that reside in the dark interior of the host hemocoel, are carnivore, and are typically free of bacterial infections. However, the conserved repertoire for these gene families may simply be due to the fact that the endoparasitoid wasp adults are free living and routinely face the fully complexity of an external environment.

The genome assembly of *M. cingulum* created an unprecedented opportunity to investigate the molecular mechanisms underlying polyembryony, the most unusual and interesting characteristics of this endoparasitoid wasp*.* The adaptive significance of this peculiar developmental mode is not yet very well understood, but one leading hypothesis suggests it is a means to increase reproductive output when females are constrained by egg production (due to very small body size) or oviposition opportunities (due to low host abundances) [6]. This includes the production of two different offspring castes, which may improve the survival rate of *M. cingulum* larvae. In polyembryonic development, one egg divides into multiple pluripotent embryos, each of which develops into an individual. The gene regulating the pluripotency of embryos at cell proliferation stage remains unclear. To this end, our analysis of miRNAs associates miR-14b with polyembryonic development, specifically by targeting regulatory genes *MBP-1*, *KMT2E* and *Runt.* Previous reports have shown that downregulation of these three target genes are essential to maintain the pluripotency of stem cell [39, 40, 47]. miR-14b is abundant in the proliferation phase during which multiple embryo cells are generated from single egg. Once the embryos and pseudogerms begin development and differentiation, expression level of miR-14b is much lower, suggesting it is sensitive and efficient during this regulatory process. The totality of available evidence collected here strongly implicates miR-14b as a factor in modulating polyembryony in *M. cingulum.* It has been reported that some long non-coding RNA were specifically expressed in either the cleavage or the subsequent primary morula stages, implying noncoding RNA might play important roles in polyembryogenesis [48].

Suppression or avoidance of host immune response is a key element of wasp biology. Active suppression strategies, such as teratocytes or injecting PDVs or VLPs, have generally received the bulk of attentions in recent research. Despite receiving less attention from researchers, passive immune-evasion strategies, as employed by *M. cingulum*, are also common among parasitoid wasps. Hemomucin is currently the only protein reported to play an important role in evading host cellular immune reactions. Our previous study proved that after the connection of the sugar chain (Gal-GalNAc) and peptide (S/T) in hemomucin were digested by O-glycosidase, more *M. cingulum*’s embryos were encapsulated compared to control [13]. We further proved that the recombinant unglycosylated hemomucin could not protect the sephadex A-25 beads mimicked parasitoid egg from being encapsulated by hemocytes of *O. furnacalis*, however, the recombinant glycosylated hemomucin could (unpublished data). Here, we found that only hemomucin in parasitoid taken passive immune evasion strategies possessed the typical mucin domain (Fig. 6). So, we speculated that when parasitoids gain more active factors such as PDVs, they do not rely on the protection of hemomucin, so the mucin domain were lost during evolution. The immune evasion function of mucin has been proved in other parasites such as *Trypanosoma cruzi* [49] and virus [50, 51], indicating mucin domain in glycoprotein, which located on the parasite’s surface, should play vital role during the co-evolution of parasite and host. However, further studies are needed to verify this hypothesis.

## Conclusions

We obtained the draft genome of parasitoid wasp *M. cingulum*. Comparative genomics analysis of 12 insects including six wasps indicated that parasitoid life has shaped the wasp genomes, resulted in the expansion or contraction of some gene families associated with chemical communication, detoxification, development, cell cycle, etc. Based on a series of analyses of *M. cingulum*’s genome and transcriptome, we found that the miR-14b might play a key role in regulating polyembryonic development of this parasitoid wasp. Only hemomucin in endoparasitoid wasps taking passive immune evasion strategies possessed the typical mucin domain. Further analysis suggested that the glycosylation of the mucin domain in these wasps might confer the immune evasion.

## Methods

### Genome sequencing

Animal experiments were approved by the Molecular Ecology and Pest Control lab, School of Life Sciences, Sun Yat-sen University. We used a whole genome shotgun strategy and the next-generation sequencing technologies on the Illumina HiSeq 2000 platform to sequence the genome of *M. cingulum*. DNA was extracted from pooled adult males. To decrease the risk of non-randomness, we built different insert sizes libraries. Three paired-end sequencing libraries of *M. cingulum* (170 bp, 500 bp and 800 bp) and 1 mate-pair large insert size sequencing library (8 Kb) were constructed respectively. In total, we got 103.67 G raw data for *M. cingulum*. After filtering out low quality and duplicated reads, 93.24 G data were maintained for assembly for *M. cingulum* (Additional file 1: Table S1).

### Transcriptome sequencing

The transcriptomes of the morula, embryo, pseudogerm, larva, female and male adult of *M. cingulum* were sequenced using Illumina 2000 platform with paired-end library. Total RNA was isolated from the sample of mixed embryos including morula and embryo, embryo, pseudogerm, larva, female adult and male adult of *M. cingulum* using TRIzol kit following the manufacturer’s protocol (Life Technologies, USA). RNA sequencing libraries were constructed using Illumina mRNA-Seq Prep Kit. Oligo (dT) magnetic beads were used to purify poly (A) containing mRNA molecules. The mRNA was fragmented and the first strand cDNA was synthesized by reverse transcription using a random primer. The second-strand cDNA was synthesized with DNA polymerase I to produce double-stranded cDNA fragments. The double stranded cDNA was end-repaired using Klenow and T4 DNA polymerases. After ligation to paired-end sequencing adapters, gel electrophoresis was used for size selection. Finally, the library preparation was completed by PCR amplification and the libraries were sequenced using Illumina HiSeq 2000 platform (101 bp at each end).

### Estimation of genome size

Genome size determinations were performed with flow cytometry following the procedures described in Hare & Johnston [52] with some modifications. A whole body of *M. cingulum* standard (1C = 175 Mbp) were placed into 1 ml of Galbraith buffer in a 2-ml Kontes Dounce homogenizer tube and stroked 15 times with the A pestle to release nuclei from both the samples. The resultant solution was filtered through 40 U nylon mesh, stained a minimum of 20 min in the dark with 25 μl of propidium iodide, and then run on a Partec Cyflow cytometer to score relative red fluorescence (> 590 nm) of nuclei from the sample and standard. The amount of DNA in the sample was determined as the mean channel number of the 2C peak of the sample divided by the mean channel number of the 2C peak of the standard times the amount of DNA in the standard. All DNA estimates were determined from a co-preparation of sample and (internal) standard. The position of the sample peak relative to that of the other peaks was established by a single run with the sample or (external) standard prepared and stained individually. *M. cingulum* males (161 Mb, 1C peak is channel 82.00). *M. cingulum* females (314 Mb, 2C peak is channel 160.00) (Additional file 1: Figure S1).

Genome size was also estimated by K-mer analysis. The distribution of K-mer depends on the characteristics of the genome and follow a Poisson’s distribution [53]. A K-mer refers to an artificial sequence division of K nucleotides iteratively from sequencing reads. To obtain independent estimates of genome size and repeat content we used the software jellyfish (version 1.1.4) to generate k-mer spectra of original raw sequencing data [54]. The size of K-mer was set to 17 in this study. Short insert size libraries (170 bp and 500 bp) were sequenced and used to estimate genome size. Based on this methodology, the genome size of *M. cingulum*, was estimated to be 135 Mb (Additional file 1: Figure S2).

### Genome assembly

1. Genomic paired and mate-pair reads were quality trimmed and filtered as described in Rödelsperger et al. 2014 [55].
2. Genomic paired-end libraries and mate-pair library were used to generate a first draft assembly with the ABySS [16] assembler (Version 1.3.7) with K-mer 64, yielding an assembly with Scaffold N50 and Contig N50 79 Kb, 30 Kb respectively.
3. Next, we used SSPACE [56] (version 2.0) with the settings (−k 5 -a 0.7 -× 1 -m 90 -o 20) for scaffolding using the mate-pair data and make the Scaffold N50 increase to 170 Kb.
4. Intra scaffold gaps were closed using the Gapclosing module (version 1.12) of the SOAP package [57], increasing the Contig N50 comes to 64 Kb.
5. Last, we used the software Rabbit [58] (version 2.6.18) with the settings (K-mer_size 16 max_occ 438) to remove the redundancy of that could not be assembled.

The final assembly comprised 5696 scaffolds spanning 132 Mb (129 Mb excluding gaps) with scaffold N50 of 192 Kb. The largest scaffold spans 1.37 Mb, and the genome-wide GC content was 35.66% (Additional file 1: Table S3). We used 500 bp non-overlapped window to analysis the distribution of GC content and CpG ratio by an in-house script (Additional file 1: Figure S3). CpG ratio is defined as P[CpG]/(P[C]*P[G]). P[C] is the frequency of C nucleotide and P[G] is the frequency of G nucleotides, while P[CpG] is the frequency of CpG dinucleotides.

### Genome quality assessment

Several statistics are used to describe the completeness and contiguity of a genome assembly, and by far the most important and widely used software is BUSCO [17, 18] (version 3.0). To run BUSCO software, we selected the inecta db 9 data sets which contains 1658 benchmarking universal single-copy orthologous genes as the library. The BUSCO software was performed with default parameters.

To compare the quality of the *M. cingulum* genome with other species, we collected all the published insect genomes (Additional file 1: Table S4), and used the same parameters and procedures to assess them. The results proved that the genome assembly of *M. cingulum* had a high quality (Additional file 1: Table S5).

### Genome annotation

The *M. cingulum* genome was annotated by the OMIGA [22] genome annotation pipeline, which is an optimized Maker-based insect genome annotation workflow.

#### Identifying repeat sequences

In the pipeline, the first step is to identifying repeat sequences, because repeats complicate genome annotation [59]. Tandem Repeats Finder (TRF) was used to search tandem repeats in the genomes [60], and Novel repeat sequences were predicted by RepeatModeler (version 1.0.7), which includes two De novo programs, RECON [61] (version 1.08) and RepeatScount [62] (version 1.0.5). Transposable elements (TEs) were predicted in the assemblies by homology searching against RepBase using RepeatMasker [19] (version 4.0.5). Both programs were used with default Parameters, yielded 24.34 Mb of repeat sequences (Additional file 1: Table S6).

### Mapping RNA-Seq raw data with the genome scaffolds

The transcriptome assembly followed the protocol described by Trapnell et al. [63]. Six transcriptomes were used to provide gene expression evidence. The RNA-Seq raw data were quality checked by Trimmomatic [64] (version 0.36) and then were mapped to the genome by the Bowtie [65] (version 2.2.5). Next, TopHat [66] (version 2.1.0) was used to determine the exon/intron junctions with the genome. Finally, Cufflinks (version 2.2.1) was used to obtain putative transcripts. We named these transcripts as the Cufflink Gene Sets. All programs were used with default parameters.

#### Re-training de novo gene prediction software

To obtain high accuracy, de novo gene prediction software must be re-trained before it can be used for genome annotation. The best training strategy is to use sufficient genes of the same species as the training dataset [67]. To collect enough genes for training, we selected transcripts with intact open reading frame (ORF) from the Cufflink genes. To further maximize sensitivity for capturing ORFs that may have functional significance, a BLAST search against a database (E-value =1e-5) of UniProtKB/Swiss-Prot proteins, and searching PFAM (E-value =1e-5) to identify protein domains. After filtered by TransDecoder software, only the genes which has a complete ORFs was included. If genes had multiple transcripts, only the longest transcript was kept for further use. Then those genes were used to re-train the prediction software Augustus [68] (version 3.1) and SNAP [69] (version 2006–07-28). For GeneMark-ET [70] (Suite 4.21), more than 10 Mb of genome sequence was used to re-train the software. The default parameters were used for training.

#### Producing an official gene sets with Maker

Homolog-based predictions, de novo predictions and transcriptome-based predictions were integrated to annotate the protein coding genes in the *M. cingulum* genome. We annotated protein-coding genes using the MAKER [71] pipeline (Version 2.31). In the MAKER pipeline, sequences of homologous proteins were from the NCBI invertebrate RefSeq. Three ab initio gene prediction programs including Augustus, SNAP and GeneMark-ET which were re-trained with *M. cingulum* transcript were used to predict coding genes. Additionally, the RNA-Seq data were mapped to the genome using TopHat, and cufflinks was used to assemble transcripts to the gene models. All gene evidence identified from above three approaches were combined by MAKER into a weighted and non-redundant consensus of gene structures. All the MAKER parameters are default settings. Finally, 11,993 genes were annotated (Additional file 1: Table S7).

#### Gene function annotation

Functional annotation of 11,993 *M. cingulum* protein coding genes was carried out by BLASTP against two integrated protein sequence databases- UniProtKB/Swiss-Prot proteins and NCBI Non-redundant protein sequences (nr). The E-value cutoff was set at 1E-5. The best 20 hits were used for annotation. Protein domains were annotated by InterProScan (version 5.21–60.0) with the panther data version 10.0. Gene Ontology (GO) analysis was carried out using the software Blast2GO, yielding 59 enriched subcategories at level 2 (Additional file 1: Figure S6). The KEGG pathway annotation were got by the BlastKOALA web server, and the level 2 categories had 42 subcategories (Additional file 1: Figure S5). Clusters of Orthologous Groups of proteins (COGs) were annotated by in-house Perl scripts (Additional file 1: Figure S4). A total of 11,485, 11,617, 9094, 7254, and 4857 genes were annotated from the reference gene set using the Swissprot, nr, InterPro, GO, KEGG databases, respectively. Six hundred eighty-eight genes were not annotated by any known databases (Additional file 1: Table S7).

#### Noncoding RNA gene annotation

We use INFERNAL [72] software searching against Rfam database of release 11.0 with e-value cutoff of 1e-5 to predict noncoding RNA (ncRNAs). Four types of ncRNAs were annotated in our analysis: transfer RNA (tRNA), ribosomal RNA (rRNA), small nuclear RNA (snRNA) and small nucleolar RNA (snoRNA). In *M. cingulum* genome, in total, 148 rRNAs, 144 tRNAs, 39 snRNAs and 16 snoRNAs were annotated (Additional file 1: Table S7).

The MapMi [24] program (version 1.5.0) was used to identify the *M. cingulum* miRNA homologs by mapping all insect miRNAs in the miRBase against the *M. cingulum* genome. The sequences of protein coding genes, repetitive elements and other classes of non-coding RNAs were removed from the genome scaffolds before mapping. All algorithms were performed with default parameters. Finally, 111 miRNAs were obtained (Additional file 1: Table S7).

### Orthologs

Orthologous groups were constructed with OrthoMCL [73] using the protein sequences of *M. cingulum*, other five Hymenoptera insects (*N. vitripennis, Ceratosolen solmsi, C. floridanum, A. mellifera* and *S. invicta*), one Coleoptera species (*Tribolium castaneum*), two Diptera species (*Drosophila melanogaster* and *Anopheles gambiae*), two Lepidoptera species (*Danaus plexippus* and *Bombyx mori*), one Hemiptera species (*Cimex lectularius*) as well as one non-insect arthropod species (*T. urticae*) (Additional file 1: Table S4). The default parameters were used, yielding 590 absolutely 1:1 orthologs from 19,318 OrthoMCL clusters using a custom Perl script (Fig. 2a). We compared the predicted genes of *M. cingulum* with other two published Hymenopteran genomes (*C. solmsi* and *N. vitripennis*) and *D. melanogaster* using OrthoMCL-DB [74] (version 9.0) with the default settings, yielding 3850 orthologs groups in four species (Fig. 2b). The distribution of pairwise amino acid identity was counted for each pair of the orthologs genes by the needle module in the EMBOSS [75] packages (version 6.6.0)(Fig. 2c).

### Synteny

To understand the potential chromosome rearrangement between *N. vitripennis*, *M. cingulum* and *C. floridanum*, best reciprocal hit of protein sequences using BLASTP with an e-value < 0.01 between any two pairs of species were defined as orthologous counterparts. The similarity of genes was indicated as a density plot of the product of aligning ration and identity. The aligning ratio was inferred by the size of aligning loci divide the size of shorter protein sequence in the alignment. The identify information was derived directly from the Blastp alignment. Synteny blocks were identified based on the orthologous gene order detected as above. Synteny blocks were defined when at least 3 orthologous counterparts were both clustered (not interrupted by more than 5 genes) and located in continuous loci in a single scaffold for each species in each pair of species (Fig. 2d).

### Phylogenetic tree and divergence time

We constructed a phylogenetic tree of *M. cingulum* and other selected insects (*A. gambiae, A. mellifera, B. mori, C. floridanum, C. solmsi, C. lectularius, D. plexippus, D. melanogaster, N. vitripennis, S. invicta, T. urticae, T. castaneum*) using 590 single-copy orthologous genes. We generated multiple sequence alignments for each 1:1 orthologs cluster using MUSCLE. The resulting alignments were trimmed using trimAl to remove positions with gaps in more than 20% of the sequences, and concatenated to one super-sequence for each species, respectively. Then we used the maximum likelihood method implemented in RAxML [26] to reconstruct the phylogenetic tree. Modeltest [76] was used to select the best substitution model. RAxML was used to reconstruct the phylogenetic tree with the aLRT method for branch support and *T. castaneum* was used as an outgroup species. The values of statistical support were obtained from 1000 replicates of bootstrap analyses. The Reltime ML [77] approach was used to estimate the species divergence time using the program MEGA [78] (version 7.0.18) and the 250 million years’ divergence time between *D. melanogaster* and *A. gambiae* [79, 80] was selected as divergence time calibration constraints that used to convert relative divergence times to absolute divergence times (Fig. 2a).

### Gene family clusters, expansion and contraction

Protein data for 13 species (*A. gambiae*, *A. mellifera*, *B. mori*, *C. floridanum*, *C. solmsi*, *C. lectularius*, *D. plexippus*, *D. melanogaster*, *M. cingulum*, *N. vitripennis*, *S. invicta*, *T. urticae*, *T. castaneum*) were downloaded from the Ensembl database or NCBI or other databases (Data source showed at Additional file 1: Table S4). For genes with alternative splicing variants, the longest transcripts were selected. We used Treefam [81] to define a gene family as a group of genes that descended from a single gene in the last common ancestor of considered species [53]. We used CAFÉ [82] to identify gene family expansions and contractions. This revealed 824 gene family expansions and 3980 gene family contractions in *M. cingulum* (Fig. 3).

### Hemomucin gene analysis

We downloaded all insects OGS from InsectBase [83]. The hemomucin protein sequences of *M. cingulum* and *D. melanogaster* were retrieved from GenBank of the National Center for Biotechnology Information (NCBI) [84] as reference sequences. The candidate hemomucin genes of each species were obtained by the BLASTP against the reference sequences with E-value 1E-30. Then the candidate hemomucin genes were analyzed using the HMMER [85] (version 3.1b2). To ensure reliability, the sequences short than 300 bp were removed.

The phylogenetic relationships of hemomucin in different insects was inferred using the Neighbor-Joining method [86]. The optimal tree with the sum of branch length of 10.49620813 was shown. The bootstrap value was set as 1000 replicates and the support values were given. The evolutionary distances were computed using the p-distance method and were in the units of the number of amino acid differences per site. The analysis involved 79 amino acid sequences. All positions containing gaps and missing data were eliminated. There was a total of 215 positions in the final dataset. Evolutionary analyses were conducted in MEGA (version 7.0.18) (Fig. 6a).

To investigate the roles of hemomucin conferring the passive evasion in wasp, we selected eight wasp species (*Microplitis bicoloratus, M. demolitor, M. cingulum, Cotesia vestalis, C. rubecula, C. glomerata, V. canescens, Diadromus collaris*) which have strong evidence of either passive evasion or parasitic factors. For *M. bicoloratus*, *C. vestalis*, *C. rubecula*, *C. glomerata*, *V. canescens* and *D. collaris* which did not have genome sequences, we downloaded the RNA-Seq raw data from the NCBI SRA database and assembled them by Trinity [87] (version 2.4.0) with default parameters.

Multiple amino acid sequence alignments were analyzed using the ClustalX multiple-alignment program. A phylogenetic tree was constructed using MEGA (version 7.0.8) based on the known amino acid sequences of hemomucin of insects. The protein pattern and profile of hemomucin genes were obtained from the PROSITE database using InterProScan. The transmembrane helix and signal peptide were analyzed using SignalP (version 4.1) and TMHMM (version 2.0). The potential O-glycosylation sites and phosphorylation sites were predicted using NetOGlyc (version4.0) and NetPHos online server (http://www.cbs.dtu.dk/services/NetOGlyc and http://www.cbs.dtu.dk/services/NetPhos/) (Fig. 6b, Fig. 6c, Additional file 1: Table S9).

### Transcriptome analyses

We used cuffdiff to analyze the differential gene expression. First, raw RNA-Seq reads were processed to remove adapter and low-quality sequences using Trimmomatic. Next, the clean reads were aligned to the assembled *M. cingulum* genome Scaffolds using bowtie. Raw counts for each predicted gene were derived from the read alignments and normalized to fragments per kilobase of exon model per million mapped fragments (FPKM) and differential expression analyses were performed using cuffdiff. The raw *P*-values were adjusted for multiple testing using the false discovery rate (FDR). For each comparison, genes with FDR < 0.05 and fold change > 2 were considered as differentially expressed genes.

### miRNA gene expression analysis and target prediction

The raw RNA-Seq reads were mapped to the extended miRNA precursors. We used an in-house Perl script to count the raw reads of each miRNA. Then, the software DEseq2 [88] was used to analyze the differential expressions of miRNA genes. For each comparison, miRNA genes with *p* < 0.05 were considered as differentially-expressed miRNA.

To predict the miRNA targets, we employed five softwares miRanda [89] (version 3.3a) with maximum free energy parameter − 25 kcal/mol, Targetscan [90] (version 7.0) with default parameters, RNAhybird [91] (version 2.1.2) with maximum free energy parameter − 20 kcal/mol, PITA [92] (version) with maximum free energy parameter − 10 kcal/mol and RNA22 (version) with default parameters. The miRNA-mRNA relations which were predicted by all five software packages were kept for further analysis.

### miRNA target validation

#### 3’UTR cloning

To obtain the 3’UTR of target genes in *M. cingulum*, 3’RACE reactions were performed using a SMARTer™ RACE cDNA Amplification kit (Clontech, Mountain View, CA, USA) according to the user’s manual. Gene-specific primers (GSP) used for 3’-RACE reactions were designed based on the coding region sequences (CDS) using PRIMER PREMIER 5.0 (Additional file 1: Table S16). The first-step PCRs were performed with the GSPs and universal primer mix. The PCR conditions were: incubation at 94 °C for 3 mins; five cycles at 94 °C for 30 s, 72 °C for 3 mins; five cycles at 94 °C for 30 s, 70 °C for 30 s, 72 °C for 3 mins; and 25 cycles at 94 °C for 30 s, 68 °C for 30 s, 72 °C for 3 mins, with a final extension of 72 °C for 10 mins. The PCR products were separated on an agarose gel and purified using the MiniBEST Agarose Gel DNA Extraction Kit (Takara, Otsu, Japan). Purified DNA fragment was ligated into the pGEM-T Easy Vector (Promega) for sequencing (BGI, Shenzhen, China).

#### Cell culture and luciferase assay

3’UTR fragments to test were cloned at the downstream of the firefly luciferase gene. pMIR-REPORT vector (Obio, Shanghai, China) was used as a firefly luciferase reporter vector, and pRL-CMV vector (Promega, Madison, WI, USA) was used as the Rellina luciferase control reporter vector. We used the HEK293T cell line (Shanghai Institutes for Biological Sciences, Shanghai, China) for the assay and the cells were cultured at 37 °C, 5% CO_2_ with DMEM (Gibco, Grand Island, NY, USA) + 10% FBS (Hyclone, logan, UT, USA) and plated in 96-well culture plates at a density of 2 × 10^6^ cells per well for 24 h’ incubation. For DNA transfection mixture, the proportion was 0.2 μg of reporter vector, 0.01 μg of control reporter and 0.25 μl lipofectamine 2000 Reagent (Invitrogen) per well. miRNA mimics were synthesized by RiboBio (Guangzhou, China) and diluted to a concentration of 100 nM. After incubated at room temperate for 5 mins, DNA and miRNA mixed with lipofectamine 2000 reagent transfection were incubated for 20 mins, respectively. After removing 50 μl culture medium per well, 25 μl DNA transfection mixture and 25 μl miRNA mixture were co-transfected for almost 6 h. We applied six replicates for each sample. At 48 h after transfection, cell lysates were prepared, and we conducted firefly luciferase activity assay using Dual-Luciferase Reporter Assay System (Promega) according to the manufacturer’s protocols with Infinite M1000 (Tecan, Switzerland). The experiment was performed in three replicates. The mean of the relative luciferase expression ratio (firefly luciferase/renilla luciferase, Luc/R-luc) of the control was set to 1. Statistics was analyzed by two-tail t-test.

#### Resistance, chemical ecology and immunity related gene family analysis

Five chemical ecology related gene families of odorant receptors (ORs), gustatory receptors (GRs), ionotropic receptors (IRs), chemosensory proteins (CSPs) and odorant binding proteins (OBPs), and the insecticide target genes of known insecticides, detoxification genes of P450s, GSTs and SNMPs were identified in the *M. cingulum* genome. First, we obtained the reference protein sequences of each gene family from the GenBank of NCBI, and manually confirmed each reference sequence. Then BLASTP was used to obtain the homolog candidate sequences with E-value 1E-5. Immune-related reference genes were retrieved from the immunodb database [93] and aligned against to different wasp species using BLASTP (E-value 1E-5). All candidate sequences were filtered by HMMER (E-value 1E-5) against with the Pfam database. Multiple sequence alignments were aligned by the MUSCLE, and conservation blocks were trimmed by the trimal software. The phylogenetic trees were constructed by the RAxML with suited Model selected by the Modeltest with bootstrap value of 1000.

## Additional file

Additional file 1: **Figure S1. **Flow cytometry estimation of the genome size for the *M. cingulum*. **Figure S2.** The distribution of 17-mer frequency in *M. cingulum* genome sequencing reads. **Figure S3.** Distribution of GC content, CpG Obs/ExpRatios of *M. cingulum*(Mcin), *N. vitripennis*(Nvit) and *A. mellifera*(Amel). **Figure S4. **COG function classification of the OGS in *M. cingulum*. **Figure S5.** KEGG pathway analysis of the OGS in *M. cingulum*. **Figure S6.** GO classification of the OGS in *M. cingulum*. **Figure S7.** Venn diagram of the homologous protein-coding genes among three wasps (*M. cingulum*, *C. solmsi*, *N. vitripennis*) and fruit fly (*D. melanogaster*). **Figure S8. **Phylogenetic relationship of CSP proteins from *A. mellifera*, *C. floridanum*, *C. solmsi*, *M. cingulum*, *N.vitripennis*, *S.invicta*. **Figure S9.** Phylogenetic relationship of GR proteins from *A. mellifera*, *C. floridanum*, *C. solmsi*, *M. cingulum*, *N.vitripennis*, *S.invicta*. **Figure S10.** Phylogenetic relationship of IR proteins from *A. mellifera*, *C. floridanum*, *C. solmsi*, *M. cingulum*, *N.vitripennis*, *S.invicta*. **Figure S11.** Phylogenetic relationship of OR proteins from *C. floridanum*, *D. melanogaster* and *M. cingulum*. **Figure S12.** Phylogenetic relationship of OBP proteins from *A. mellifera*, *C. floridanum*, *C. solmsi*, *M. cingulum*, *N.vitripennis*, *S.invicta*. **Figure S13. **Phylogenetic relationship of SNMP proteins from *A.mellifera*, *C. floridanum*, *C. solmsi*, *M. cingulum*, *N.vitripennis*, *S.invicta*. **Figure S14. **Phylogenetic relationship of GST proteins from *A. mellifera*, *C. floridanum*, *C. solmsi*, *M. cingulum*, *N.vitripennis*, *S.invicta*. **Figure S15.** Phylogenetic relationship of P450 proteins from *N. vitripennis*, *D. melanogaster* and *M. cingulum*. **Figure S16.** Phylogenetic relationship of ABC proteins from *M. cingulum* and *D. melanogaster*. **Figure S17.** Different expression levels of miR-14b in different developmental stages of *M. cingulum*.**Table S1.** Genome sequencing data of *M. cingulum*. **Table S2.** Estimation of *M. cingulum* genome size using K-mer analysis. **Table S3.** Summary of the *M. cingulum* genome assembly. **Table S4.** The published insect genomes. **Table S5.** The genome assembly assessment on different insects. **Table S6.** Classification of repeat sequences identified in the *M. cingulum* genome. **Table S7.** Genome features of the *M. cingulum*, *N. vitripennis* and *A. mellifera*. **Table S8.** Gene features of *M. cingulum*,* N. vitripennis* and *A. mellifera*. **Table S9.** The insects with OGSs in InsectBase. **Table S10.** Hemomucin genes in eight wasps. **Table S11.** The different gene expression of embryo and pseudogerm transcriptomes in KEGG pathway. **Table S12.** The differently expressed miRNAs in embryo and mixed embryo transcriptomes. **Table S13.** Comparison of gene numbers for chemoreception in *A.mellifera*, *C. floridanum*, *C. solmsi*, *M. cingulum*, *N. vitripennis* and *S. invicta*. **Table S14.** Comparison of gene numbers for Gene families associated with insecticide resistance and detoxification in *D. melanogaster*, *A. mellifera*, *C. floridanum*, *C. solmsi*, *M. cingulum*, *N. vitripennis* and *S. invicta*. **Table S15.** Comparison of gene numbers of insect immune in *A. mellifera*, *C. floridanum*,* C. solmsi*, *M. cingulum*, *N. vitripennis* and *S. invicta*. **Table S16.** The PCR primer for target genes of mci-miR-14b. (PDF 6076 kb) 

## Abbreviations

ARF: ADP ribosylation factors
BUSCO: Benchmarking Universal Single-Copy Orthologs
EHMT: Euchromatic histone-lysine N-methyltransferase
GLG: Golgi apparatus protein
GST: Glutathione S-transferases
KMT2E: Histone-lysine N-methyltransferase 2E
MBP-1: c-myc promoter-binding protein 1
McHEM: *M. cingulum* hemomucin protein
miRNA: microRNA
OBP: Odorant binding proteins
OGS: Official gene set
OMIGA: Optimized Maker-based Insect Genome Annotation
PDV: Polydnaviruses
qPCR: Quantitative PCR
rRNA: Ribosome RNA
snoRNA: Small nucleolar RNA
snRNA: Small nuclear RNA
tRNA: Transfer RNA
