# Supplementary material for: The genomic features of parasitism, Polyembryony and immune evasion in the endoparasitic wasp Macrocentrus cingulum
Source: BMC Genomics. 2018 May 30;19:420. doi: 10.1186/s12864-018-4783-x (PMC5977540; doi:10.1186/s12864-018-4783-x)
Supplement: Supplementary file 1 — Figure S1. Flow cytometry estimation of the genome size for the M. cingulum. Figure S2. The distribution of 17-mer frequency in M. cingulum genome sequencing reads. Figure S3. Distribution of GC content, CpG Obs/ExpRatios of M. cingulum(Mcin), N. vitripennis(Nvit) and A. mellifera(Amel). Figure S4. COG function classification of the OGS in M. cingulum. Figure S5. KEGG pathway analysis of the OGS in M. cingulum. Figure S6. GO classification of the OGS in M. cingulum. Figure S7. Venn diagram of the homologous protein-coding genes among three wasps (M. cingulum, C. solmsi, N. vitripennis) and fruit fly (D. melanogaster). Figure S8. Phylogenetic relationship of CSP proteins from A. mellifera, C. floridanum, C. solmsi, M. cingulum, N.vitripennis, S.invicta. Figure S9. Phylogenetic relationship of GR proteins from A. mellifera, C. floridanum, C. solmsi, M. cingulum, N.vitripennis, S.invicta. Figure S10. Phylogenetic relationship of IR proteins from A. mellifera, C. floridanum, C. solmsi, M. cingulum, N.vitripennis, S.invicta. Figure S11. Phylogenetic relationship of OR proteins from C. floridanum, D. melanogaster and M. cingulum. Figure S12. Phylogenetic relationship of OBP proteins from A. mellifera, C. floridanum, C. solmsi, M. cingulum, N.vitripennis, S.invicta. Figure S13. Phylogenetic relationship of SNMP proteins from A.mellifera, C. floridanum, C. solmsi, M. cingulum, N.vitripennis, S.invicta. Figure S14. Phylogenetic relationship of GST proteins from A. mellifera, C. floridanum, C. solmsi, M. cingulum, N.vitripennis, S.invicta. Figure S15. Phylogenetic relationship of P450 proteins from N. vitripennis, D. melanogaster and M. cingulum. Figure S16. Phylogenetic relationship of ABC proteins from M. cingulum and D. melanogaster. Figure S17. Different expression levels of miR-14b in different developmental stages of M. cingulum.Table S1. Genome sequencing data of M. cingulum. Table S2. Estimation of M. cingulum genome size using K-mer analysis. Table S3. Summary of the [file 12864_2018_4783_MOESM1_ESM.pdf]

Supplementary Figures

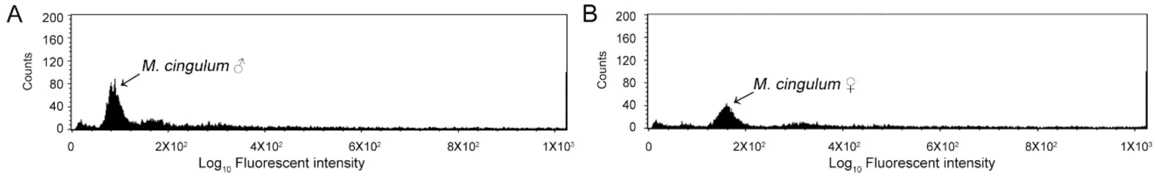

Figure S1. Flow cytometry estimation of the genome size for the *M. cingulum*. A) The Flow cytometry estimation of the male. B) Flow cytometry estimation of the female.

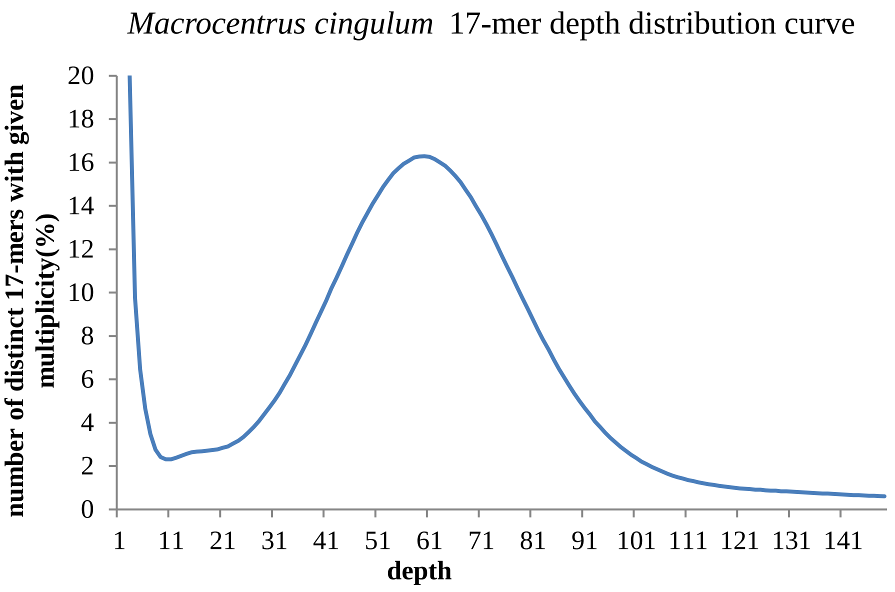

Figure S2. The distribution of 17-mer frequency in *M. cingulum* genome sequencing reads

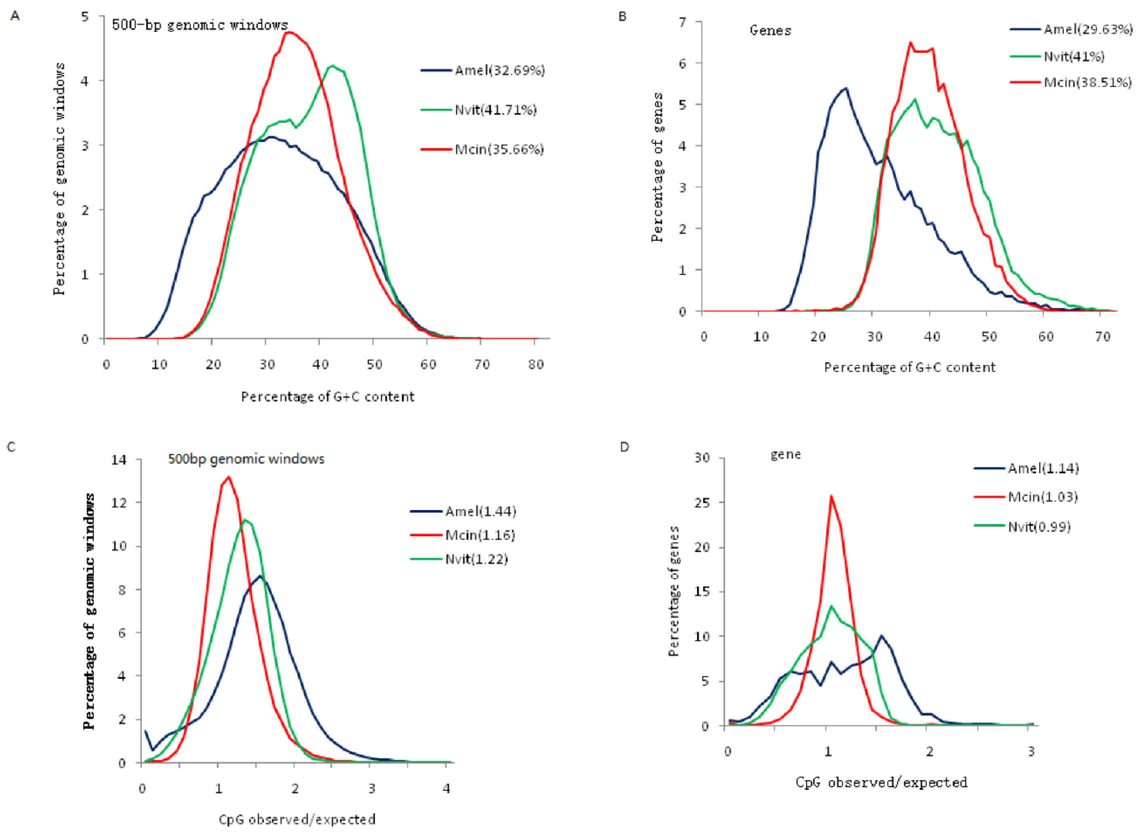

Figure S3. Distribution of GC content, CpG Obs/ExpRatios of *M. cingulum*(Mcin)*, N. vitripennis*(Nvit) and *A. mellifer*(Amel)

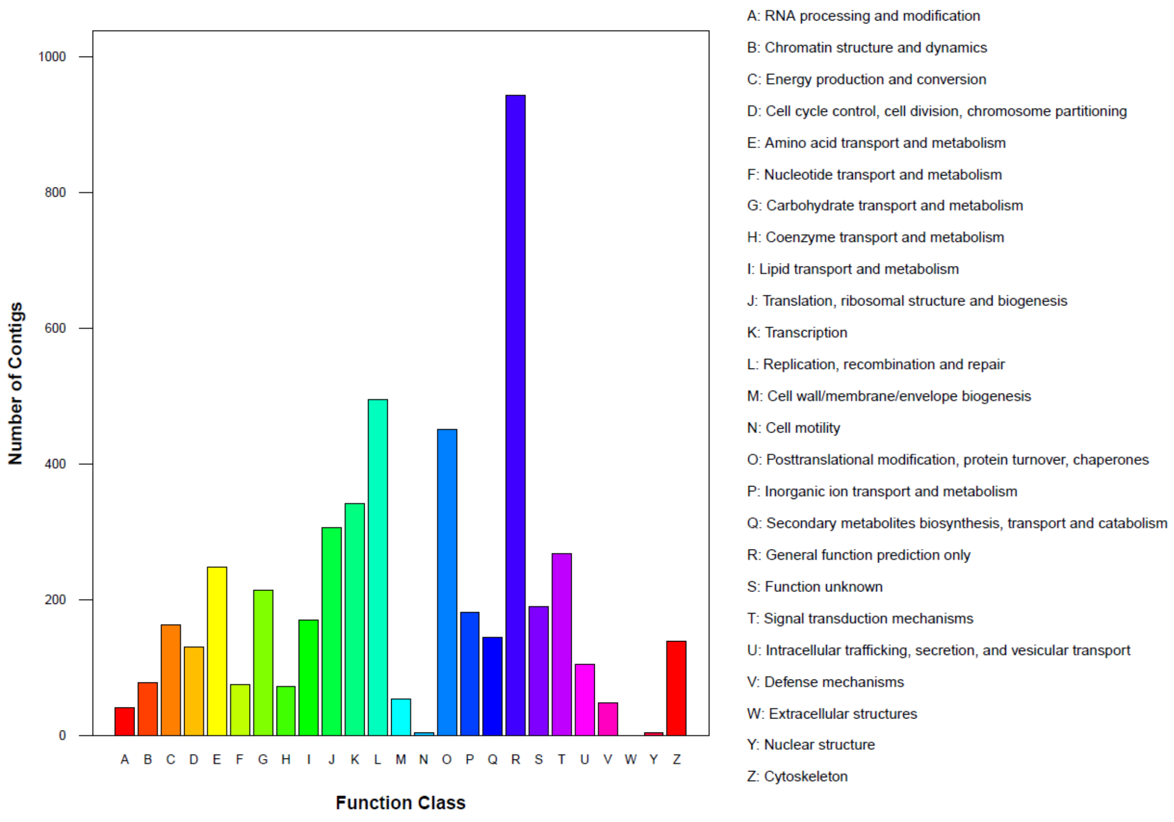

Figure S4. COG function classification of the OGS in *M. cingulum*

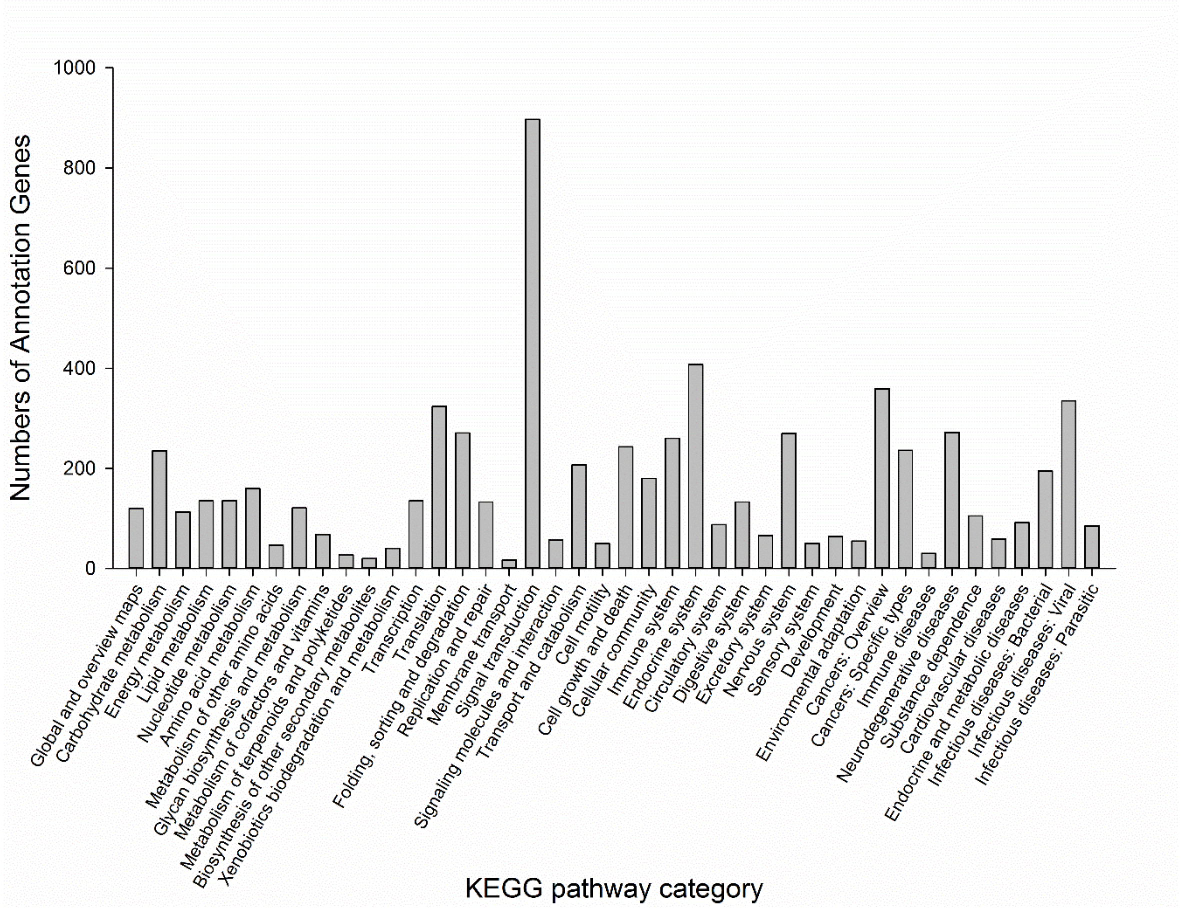

Figure S5. KEGG pathway analysis of the OGS in *M. cingulum*

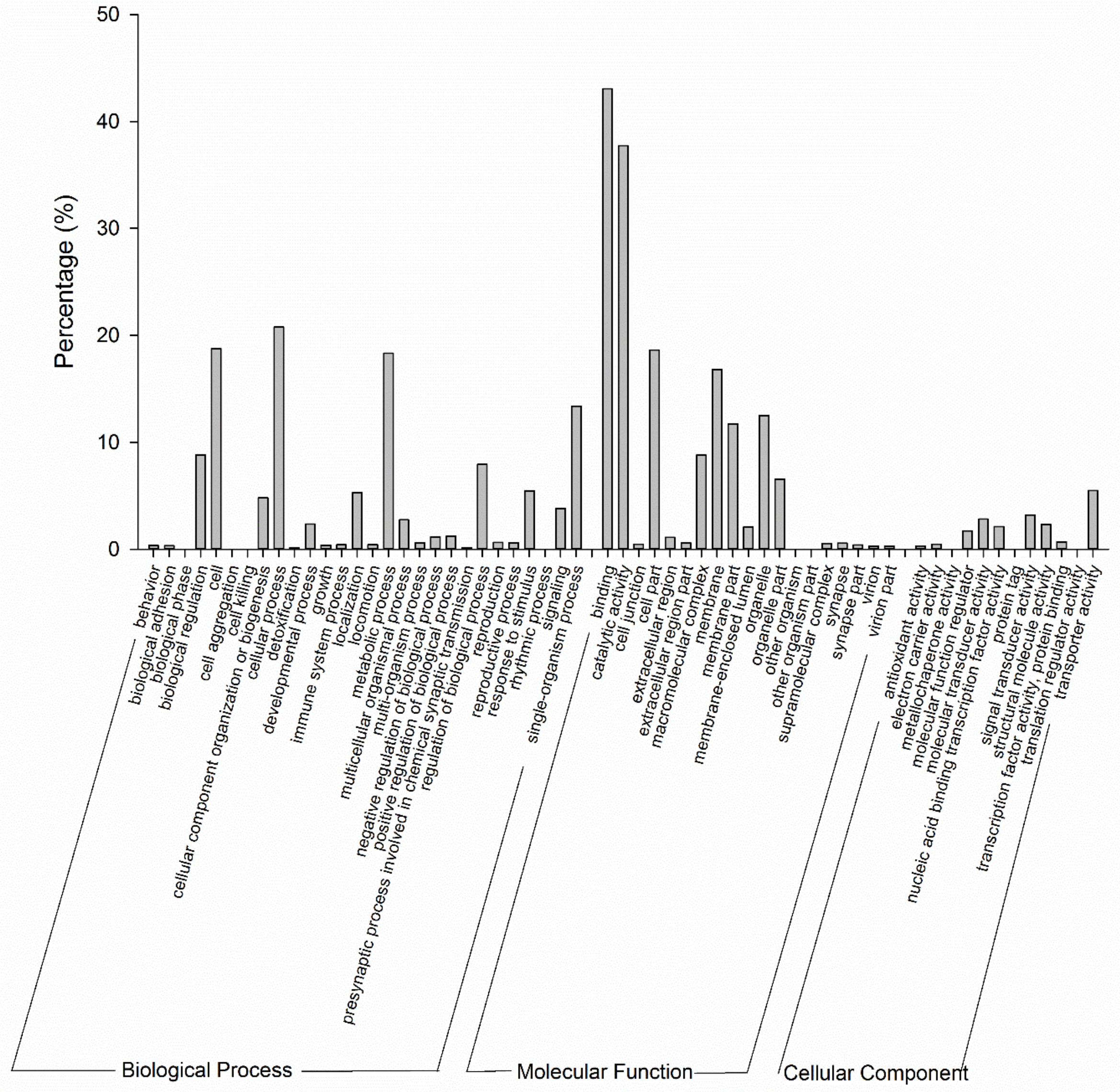

Figure S6. GO classification of the OGS in *M. cingulum*

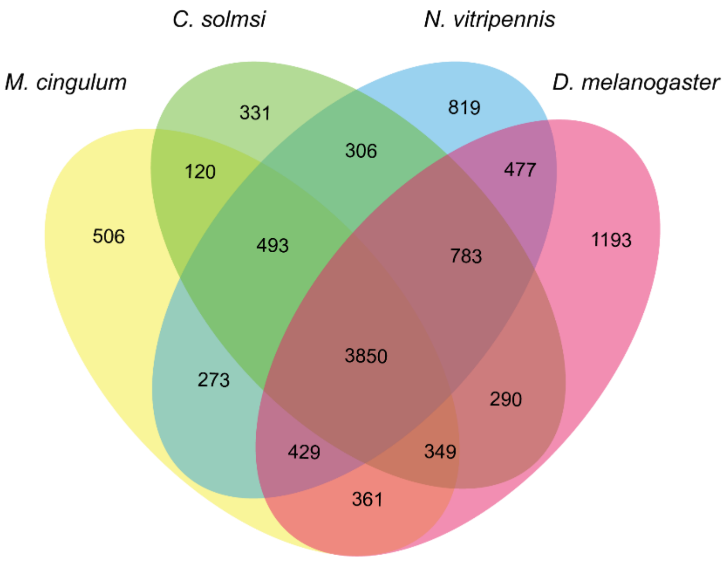

Figure S7. Venn diagram of the homologous protein-coding genes among three wasps (*M. cingulum, C. solmsi, N. vitripennis*) and fruit fly (*D. melanogaster*)

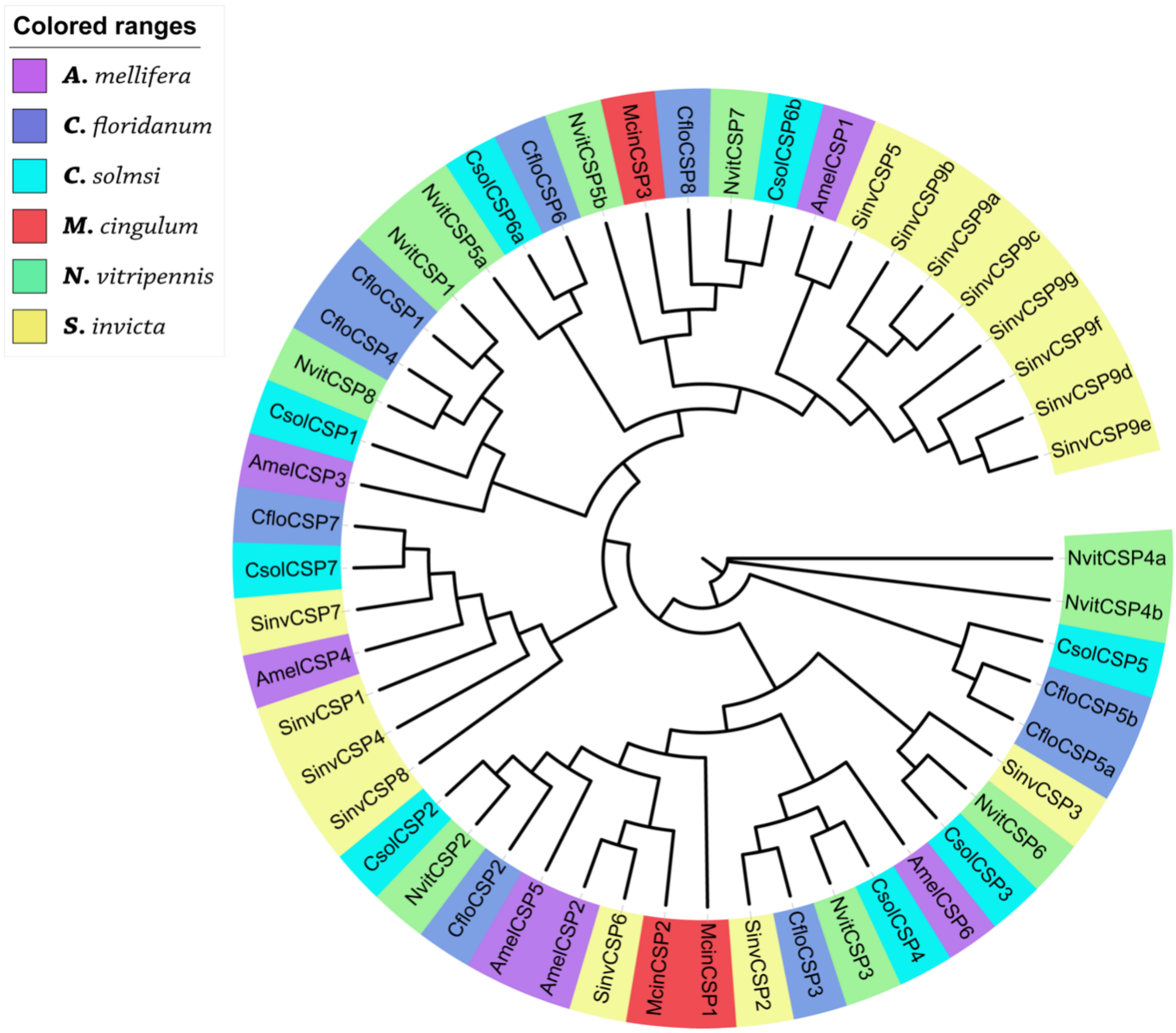

Figure S8. Phylogenetic relationship of CSP proteins from *A. mellifera*, *C. floridanum*, *C. solmsi*, *M. cingulum*, *N.vitripennis*, *S.invicta*

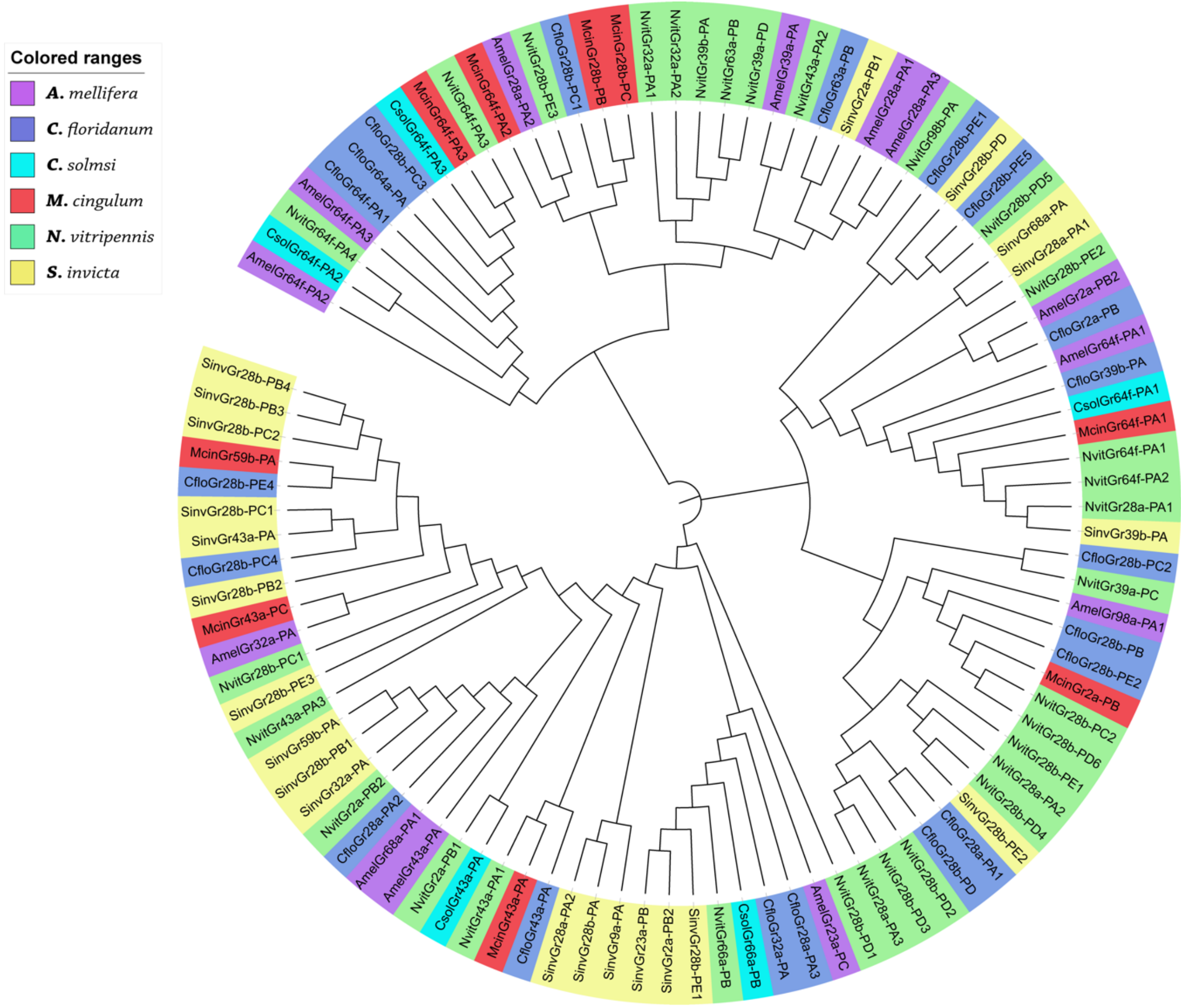

Figure S9. Phylogenetic relationship of GR proteins from *A. mellifera*, *C. floridanum*, *C. solmsi*, *M. cingulum*, *N.vitripennis*, *S.invicta*

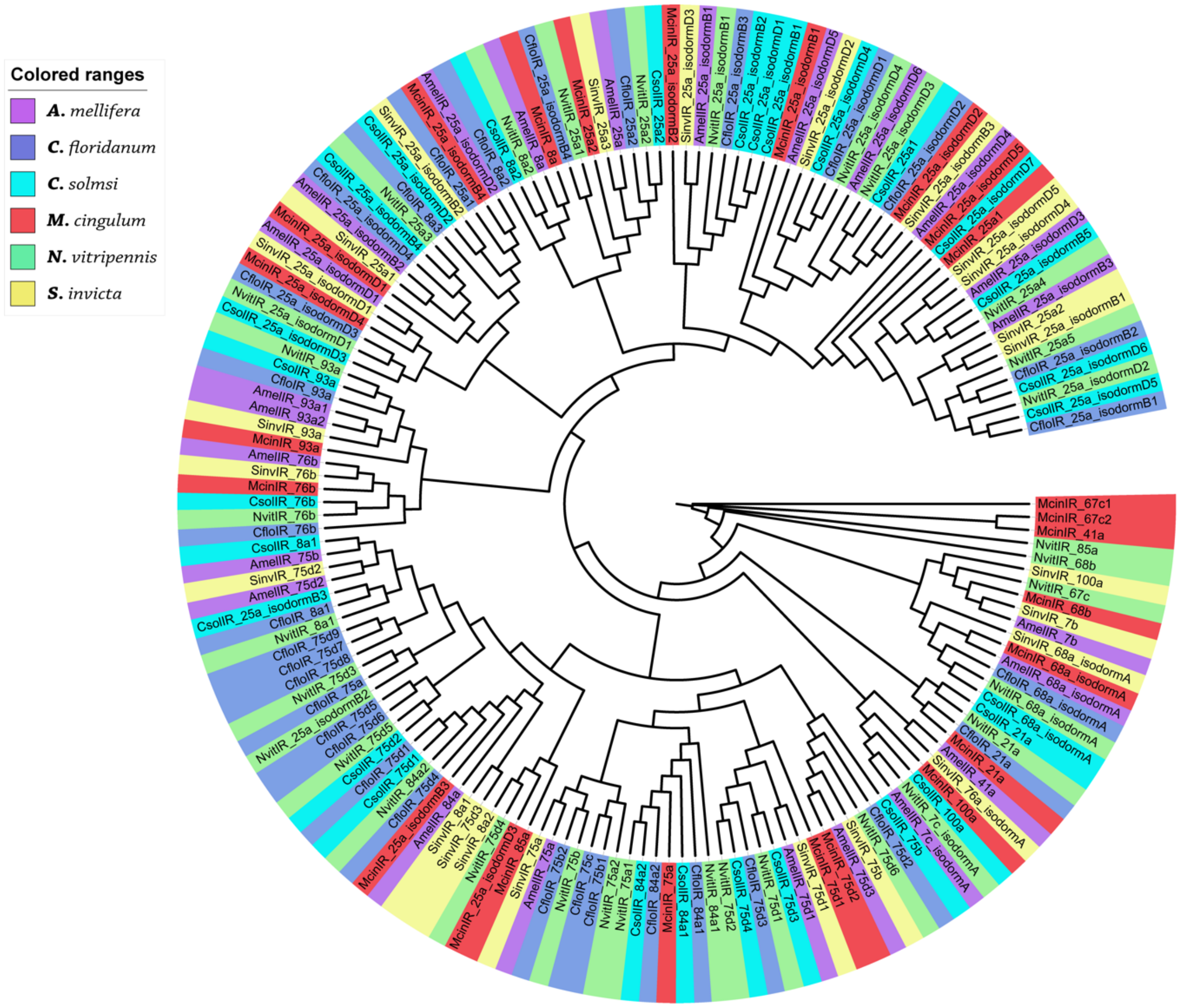

Figure S10. Phylogenetic relationship of IR proteins from *A. mellifera*, *C. floridanum*, *C. solmsi*, *M. cingulum*, *N.vitripennis*, *S.invicta*

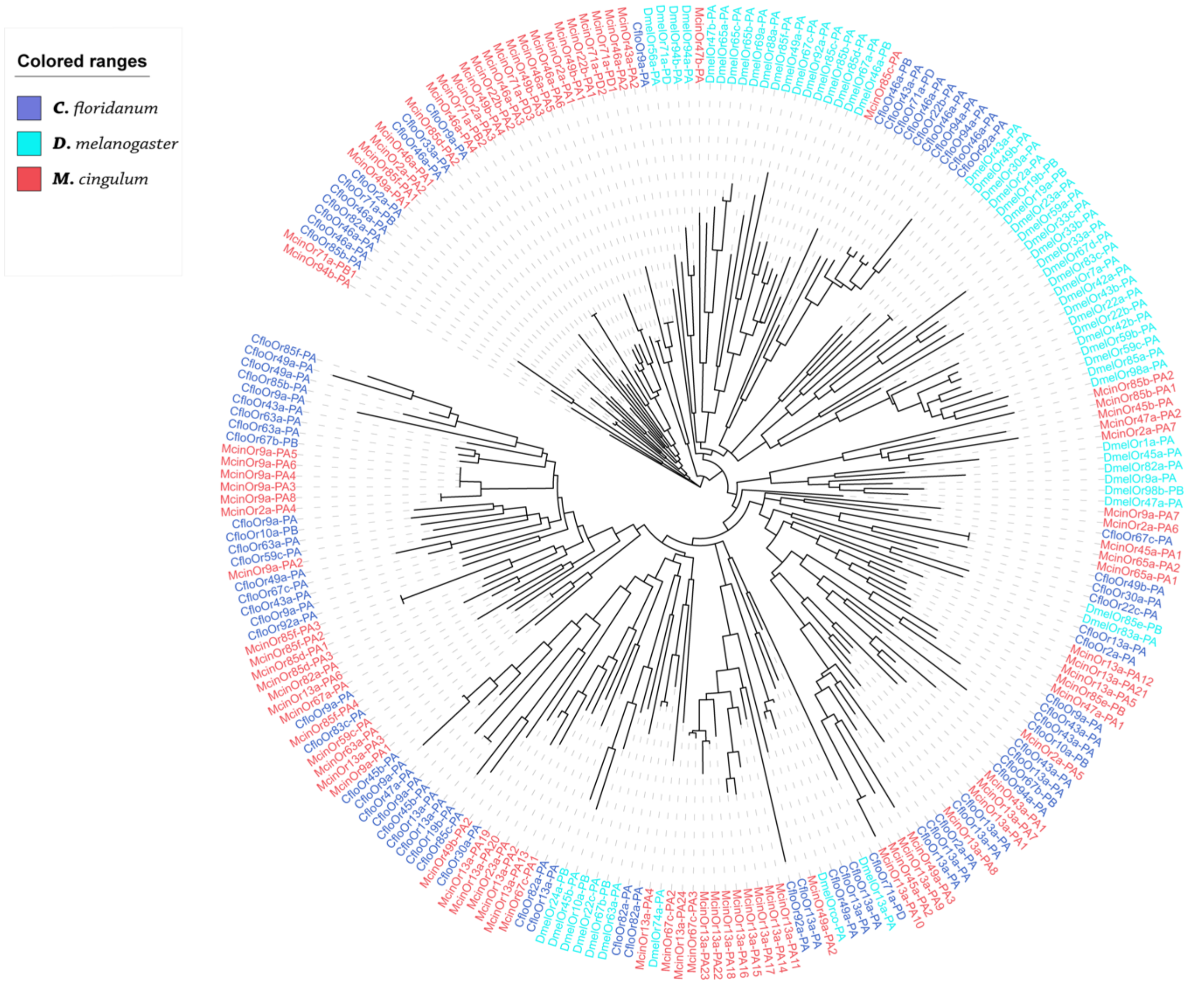

Figure S11. Phylogenetic relationship of OR proteins from *C. floridanum*, *D. melanogaster* and *M. cingulum*

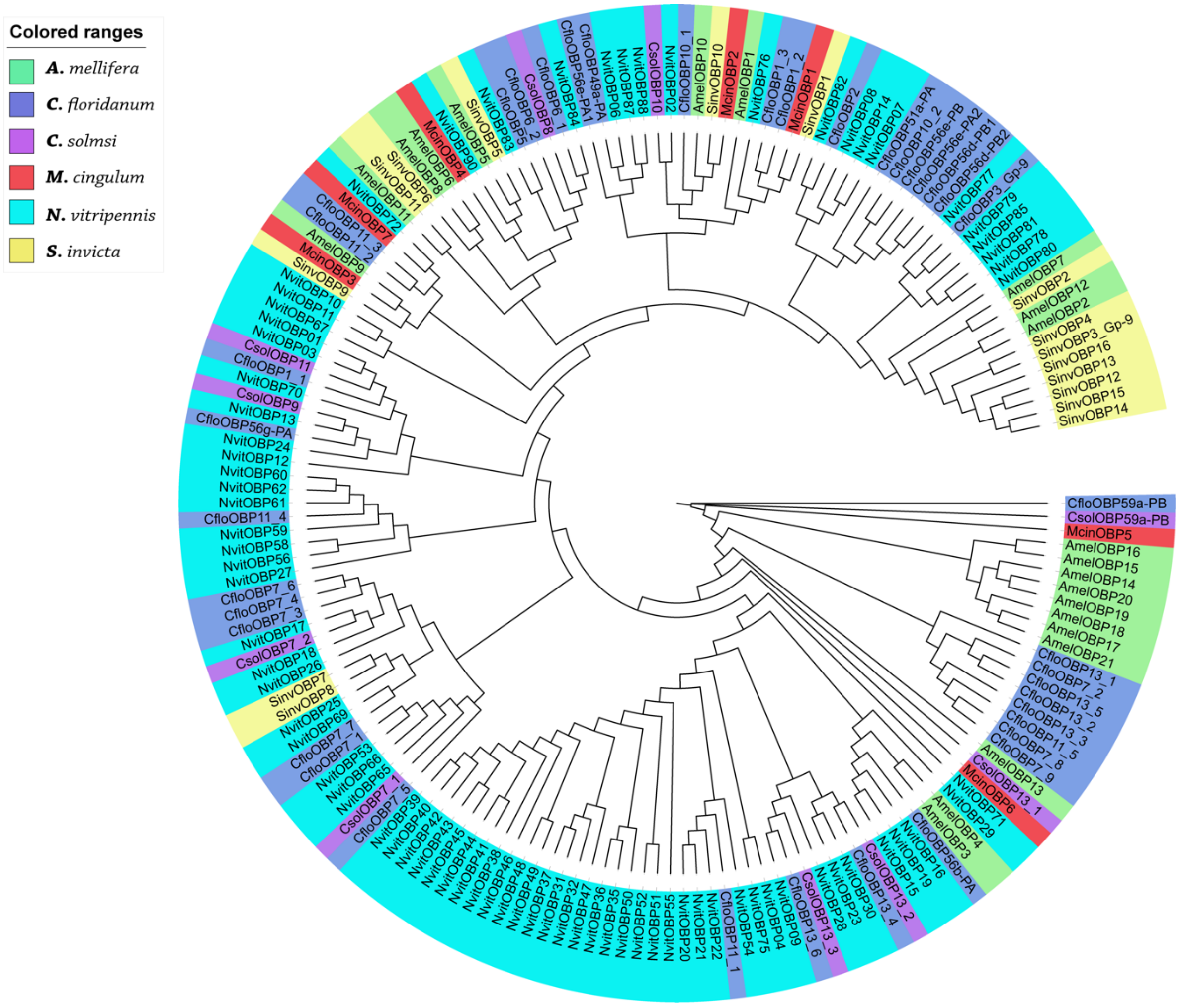

Figure S12. Phylogenetic relationship of OBP proteins from *A. mellifera*, *C. floridanum*, *C. solmsi*, *M. cingulum*, *N.vitripennis*, *S.invicta*

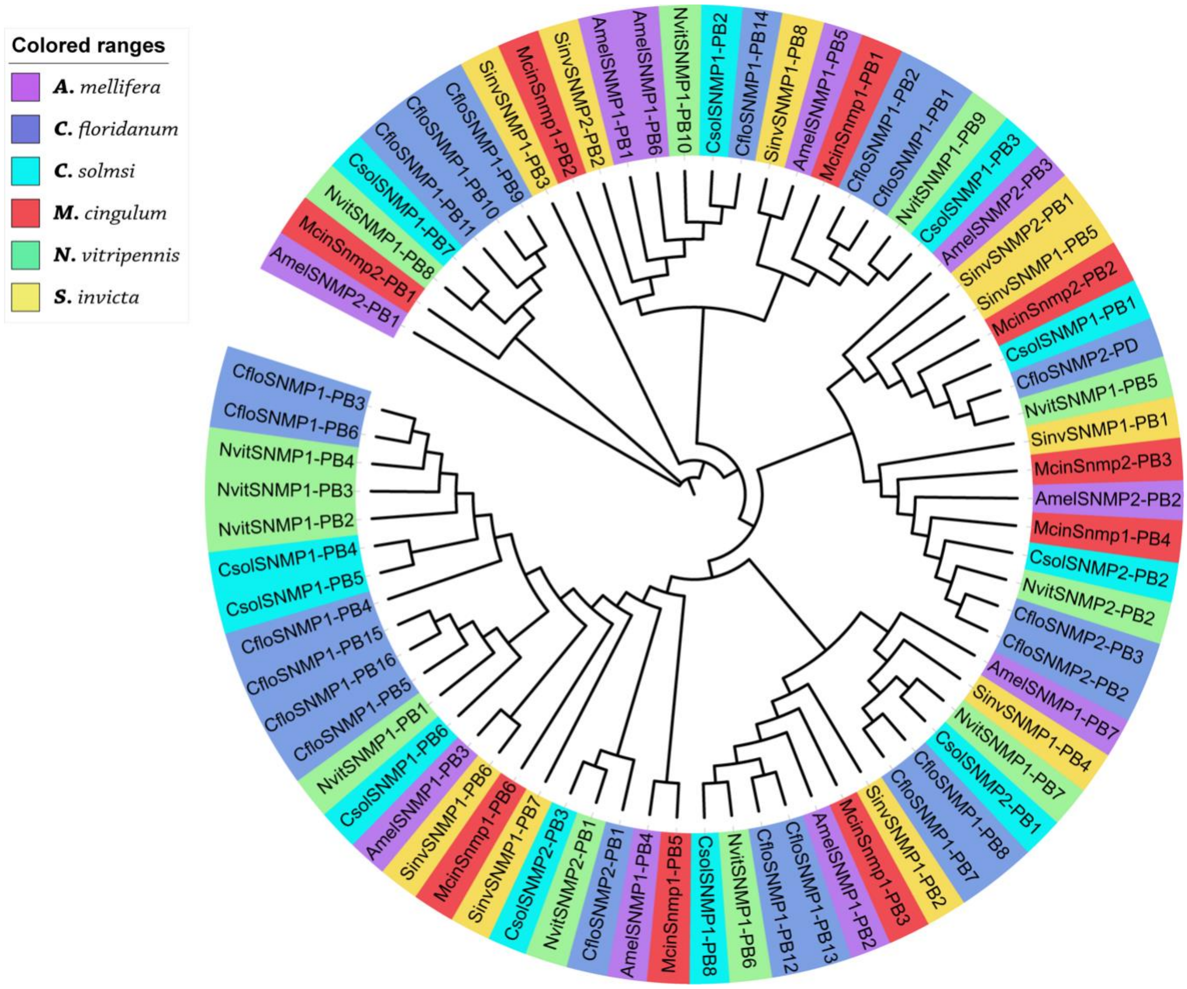

Figure S13. Phylogenetic relationship of SNMP proteins from *A. mellifera*, *C. floridanum*, *C. solmsi*, *M. cingulum*, *N.vitripennis*, *S.invicta*

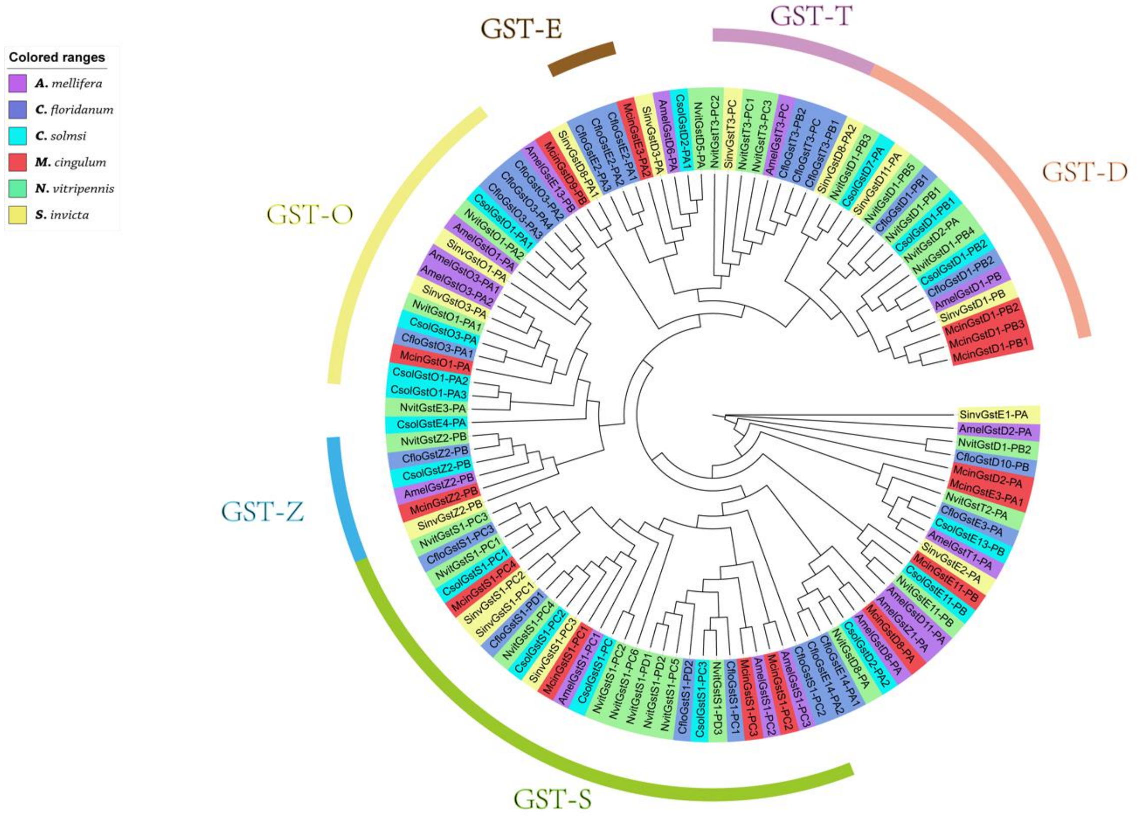

Figure S14. Phylogenetic relationship of GST proteins from *A. mellifera*, *C. floridanum*, *C. solmsi*, *M. cingulum*, *N.vitripennis*, *S.invicta*

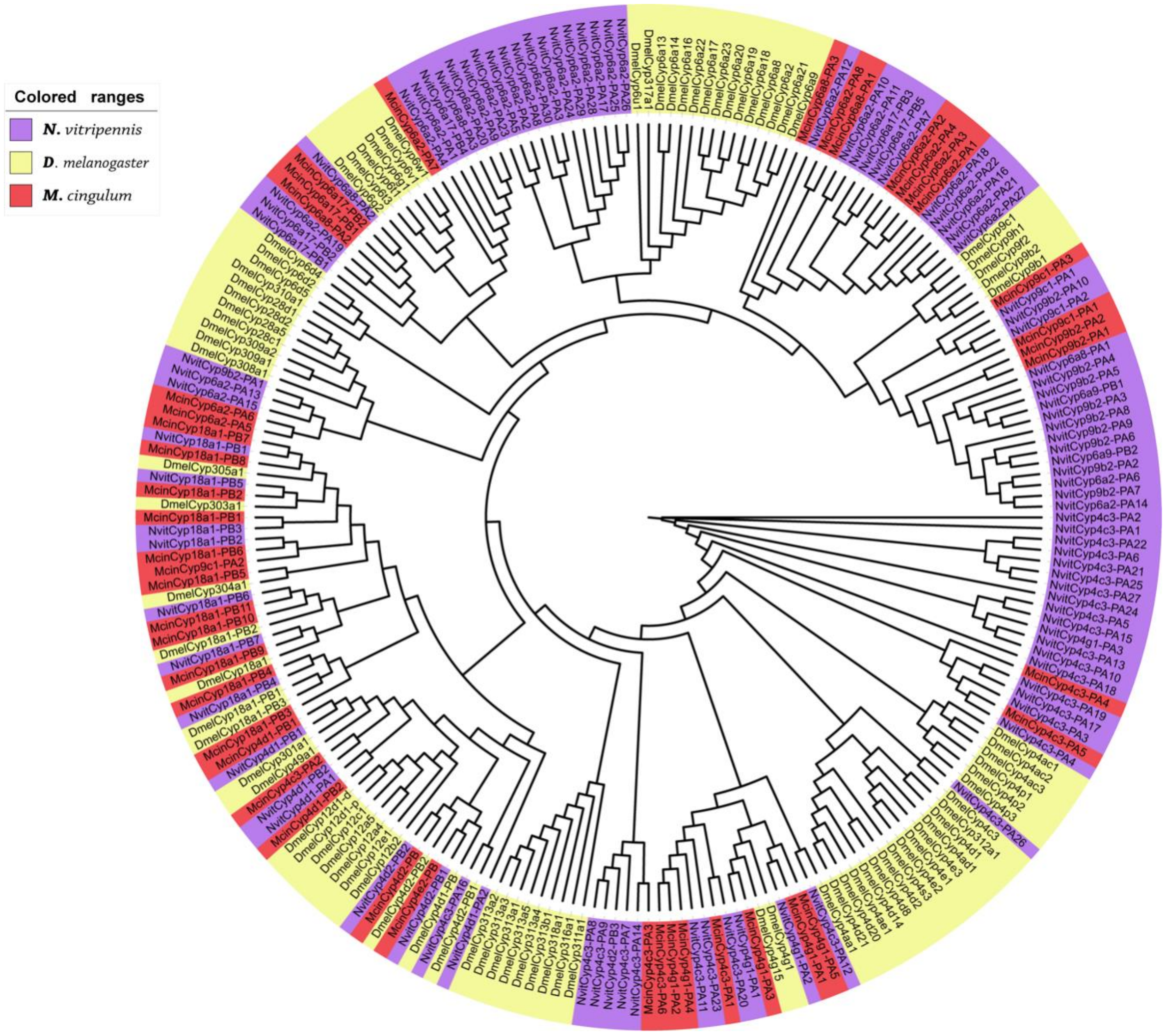

Figure S15. Phylogenetic relationship of P450 proteins from *N. vitripennis*, *D. melanogaster* and *M. cingulum*

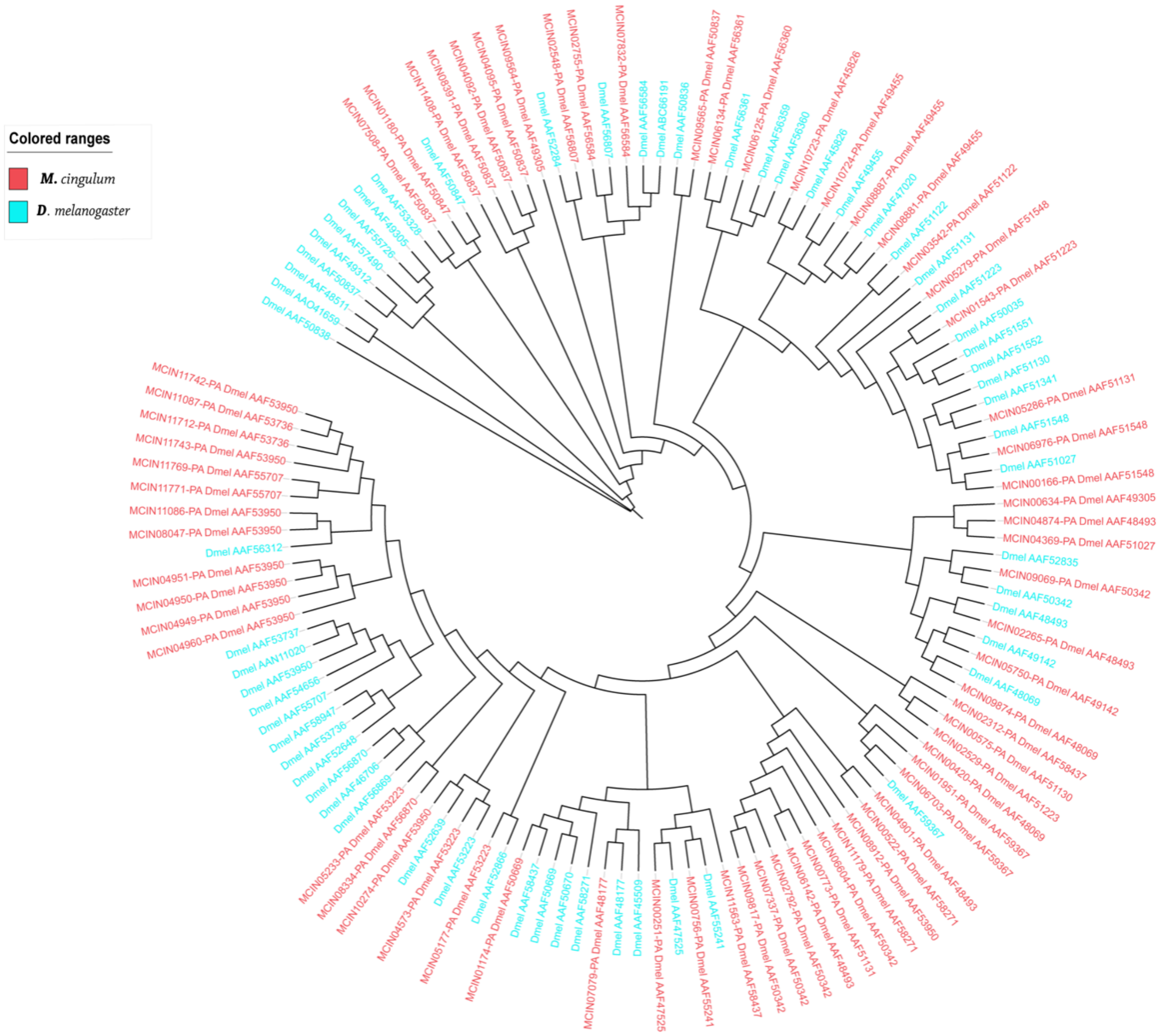

Figure S16. Phylogenetic relationship of ABC proteins from *M. cingulum* and *D. melanogaster*

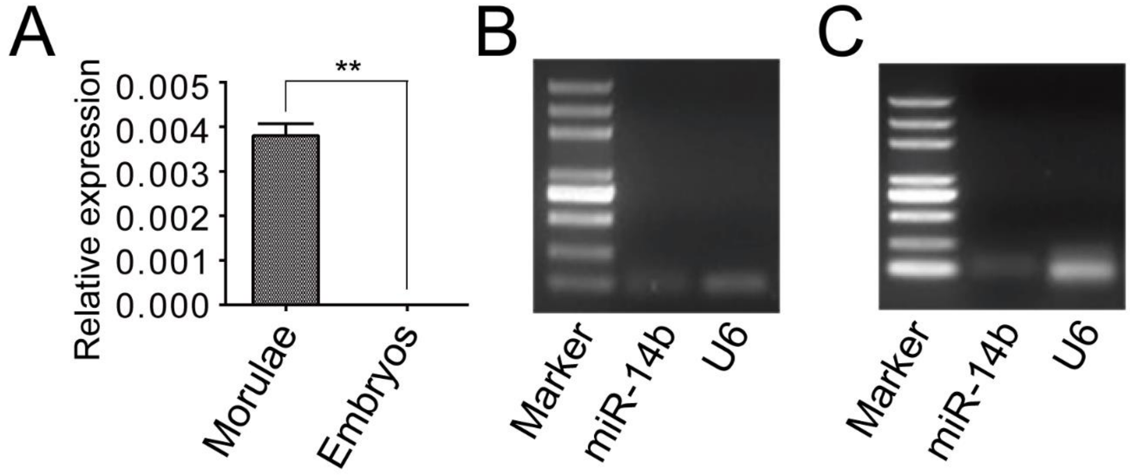

Figure S17. Different expression levels of miR-14b in different developmental stages of *M. cingulum*. A) The relative expression level of miR-14b in morulae and embryos. miR-14b is highly expressed in morulae. B) Results of quantitative real time PCR (qPCR) of miR-14b in morulae. C) Results of qPCR of miR-14b in embryos.

Supplementary Tables

Table S1. Genome sequencing data of *M. cingulum*

Library	size	Raw-data(Coverage)	Clean-data (Gb)	Read length (bp)
Paired-end	170bp	22.60Gb(167X)	20.78	101
Paired-end	500bp	30.52Gb(226X)	27.49	101
Paired-end	800bp	26Gb(191X)	23.61	101
Mate-pair	8Kb	24.55Gb(167X)	21.36	101

Table S2. Estimation of *M. cingulum* genome size using K-mer analysis

K-mer	K-mer number	K-mer depth	Genome size (bp)	Used bases (Gb)	Sequce depth (X)
17	203,535	64.4926	135,068,848	10,332,487,725	76.4979

Table S3. Summary of the *M. cingulum* genome assembly

Category	Contigs	Scaffolds
Total length (bp)	128,805,614	132,362,060
Max length (bp)	520,724	1,374,378
Average length (bp)	9,662	23,237
N20 length	146,572	458,231
N50 length (bp)	64,884	192,445
N90 length (bp)	6,290	21,264
Total sequence numbers	13,289	5,696

Table S4. The published insect genomes

Insects	GenBank	Source	References
Drosophila melanogaster	JSAE00000000	flybase	Science, 2000, 287(5461): 2185-95
Anopheles gambiae	ABKQ00000000	vectorBase	Science, 2002, 298(5591): 129-49
Bombyx mori	BABU00000000	silkDB v2.0	Science, 2004, 306(5703): 1937-40
Apis mellifera	JSUV00000000	beebase V4.5	Nature, 2006, 443(7114): 931
Aedes aegypti	AAGE00000000	vectorBase	Science, 2007, 316(5832): 1718-23
Tribolium castaneum	AAJJ00000000	Ensembl release22	Nature, 2008, 452(7190): 949-55
Culex quinquefasciatus	AAWU00000000	vectorBase	Science, 2010, 330(6000): 86-8
Camponotus floridanus	AEAB00000000	Ant Genomes Portal	Science, 2010, 329(5995): 1068-71
Harpegnathos saltator	AEAC00000000	Ant Genomes Portal	
Acyrthosiphon pisum	ABLF00000000	aphidbase V2.1b	PLoS Biol, 2010, 8(2): e1000313
Pediculus humanus	AAZO00000000	vectorBase	PNAS, 2010, 107(27): 12168-12173
Nasonia vitripennis	AAZX00000000	NasoniaBase v1.2	Science, 2010, 327(5963): 343-8
Acromyrmex echinatior	AEVX00000000	Ant Genomes Portal	Genome Res, 2011, 21(8): 1339-48
Linepithema humile	ADOQ00000000	Ant Genomes Portal	PNAS, 2011, 108(14): 5673-8
Pogonomyrmex barbatus	ADIH00000000	Ant Genomes Portal	PNAS, 2011, 108(14): 5667-72
Atta cephalotes	ADTU00000000	Ant Genomes Portal	PLoS genetics, 2011, 7(2): e1002007

Solenopsis invicta	AEAQ00000000	Ant Genomes Portal	PNAS, 2011, 108(14): 5679-84
Danaus plexippus	AGBW00000000	MonachBase v2.0	Cell, 2011, 147(5): 1171-1185
Heliconius melpomene	CAFA00000000	Heliconius Genome project	Nature, 2012
Dendroctonus ponderosae	APGL00000000	Ensembl release22	Genome Biol, 2013, 14(3): R27
Lasioglossum albipes	ANOB01000000	GCA_000346575.1	Genome Biol, 2013, 14(12): R142
Plutella xylostella	AHIO00000000	DBM-DB v1.0	Nature genetics, 2013, 45(2): 220-225
Ceratosolen solmsi	ATAC00000000	GCF_000503995.1	Genome Biol, 2013, 14(12): R141
Antarctic midge (Belgica antarctica)	JPYR00000000	GCA_000775305.1	Nat Commun, 2014, 54611
Cerapachys biroi	JASI00000000	GCF_000611835.1	Curr Biol, 2014, 24(4): 451-8
Musca domestica	AQPM00000000	GCF_000371365.1	Genome Biol, 2014, 15(10): 466
Stick insect (Timema cristinae)	MSSY02000000	Nosil Lab	Science, 2014, 344(6185): 738-42
Zootermopsis nevadensis	AUST00000000	GCA_000696155.1	Nat Commun, 2014, 53636
Locusta migratoria	AVCP00000000	GCA_000516895.1	Nat Commun, 2014, 52957
Nilaparvata lugens	AOSB00000000	OMIGA annotation project	Genome Biol, 2014, 15(12): 521
Spodoptera frugiperda	JQCY00000000	GCA_000753635.2	Genomics, 2014, 104(2): 134-43
Diuraphis noxia	MPKX01000000	GCF_001186385.1	BMC Genomics, 2015, 16(1): 429
Hypothenemus hampei	LBGY00000000	GCA_001012855.1	Rep, 2015, 512525
Lucilia cuprina	JHUJ00000000	GCA_000699065.1	Nat Commun, 2015, 67344

Operophtera brumata	JTDY01000000	GCA_001266575.1	Genome Biol Evol, 2015, 7(8): 2321-32
Cimex lectularius	JHUN00000000	GCA_000648675.1	Nat Commun, 2016, 710165
Ceratitis capitata	AOHK00000000	GCF_000347755.1	Genome Biol, 2016, 17(1): 192

Table S5. The genome assembly assessment on different insects

Insects	BUSCO				
	Complete	Single	Duplication	Fragment	Missing
Drosophila melanogaster	0.996984	0.989747	0.00723764	0.001809	0.001206
Anopheles gambiae	0.995175	0.977081	0.01809409	0.001206	0.003619
Bombyx mori	0.95778	0.953559	0.00422195	0.018697	0.023522
Apis mellifera	0.971049	0.968034	0.00301568	0.014475	0.014475
Aedes aegypti	0.972256	0.897467	0.0747889	0.010253	0.017491
Tribolium castaneum	0.993366	0.990953	0.00241255	0.004222	0.002413
Culex quinquefasciatus	0.967431	0.917973	0.04945718	0.007841	0.024729
Camponotus floridanus	0.923402	0.916164	0.00723764	0.016285	0.060314
Harpegnathos saltator	0.586852	0.582027	0.00482509	0.001809	0.411339
Acyrthosiphon pisum	0.768396	0.731604	0.03679131	0.04041	0.191194
Pediculus humanus	0.872135	0.870326	0.00180941	0.009047	0.118818
Nasonia vitripennis	0.95959	0.950543	0.00904704	0.020507	0.019903
Acromyrmex echinatior	0.472256	0.471653	0.00060314	0.002413	0.525332
Linepithema humile	0.962002	0.957177	0.00482509	0.007841	0.030157
Pogonomyrmex barbatus	0.972859	0.970446	0.00241255	0.0193	0.007841

Atta cephalotes	0.973462	0.965621	0.00784077	0.013872	0.012666
Solenopsis invicta	0.962002	0.958987	0.00301568	0.021713	0.016285
Danaus plexippus	0.924005	0.905911	0.01809409	0.017491	0.058504
Heliconius melpomene	0.956574	0.951146	0.00542823	0.018094	0.025332
Dendroctonus ponderosae	0.930639	0.875151	0.05548854	0.028347	0.041013
Lasioglossum albipes	0.989144	0.969843	0.01930036	0.006634	0.004222
Plutella xylostella	0.878166	0.727986	0.15018094	0.026538	0.095296
Ceratosolen solmsi	0.375754	0.375151	0.00060314	0	0.624246
Antarctic midge (Belgica antarctica)	0.979493	0.974065	0.00542823	0.013269	0.007238
Cerapachys biroi	0.983112	0.970446	0.01266586	0.01146	0.005428
Musca domestica	0.987334	0.969843	0.01749095	0.004222	0.008444
Stick insect (Timema cristinae)	0.929433	0.924005	0.00542823	0.036791	0.033776
Zootermopsis nevadensis	0.886007	0.875754	0.01025332	0.001809	0.112183
Locusta migratoria	0.404704	0.387214	0.01749095	0.283474	0.311821
Nilaparvata lugens	0.854041	0.770808	0.08323281	0.033776	0.112183
Spodoptera frugiperda	0.781665	0.772618	0.00904704	0.062726	0.155609
Diuraphis noxia	0.642943	0.627865	0.01507841	0.042823	0.314234
Hypothenemus hampei	0.966224	0.963812	0.00241255	0.021713	0.012063

Lucilia cuprina	0.961399	0.945115	0.01628468	0.00965	0.028951
Operophtera brumata	0.946321	0.913148	0.0331725	0.03076	0.022919
Cimex lectularius	0.932449	0.908926	0.02352232	0.016888	0.050663
Ceratitis capitata	0.991556	0.987334	0.00422195	0.003619	0.004825
Macrocentrus cingulum	0.98854	0.941496	0.04704463	0.006031	0.005428

Table S6. Classification of repeat sequences identified in the *M. cingulum* genome

Repeat types		Number of elements	Length occupied (bp)	Percentages of sequence (%)
Interspersed repeats	SINE	236	67,958	0.1
	LINE	1,101	423,211	0.32
	LTR	5,229	1,465,193	1.11
	DNA elements	9,034	3,180,053	2.41
	Unclassified	59,557	15,510,372	11.71
Satellites		2	1,283	0
Simple repeats		47,377	1,843,813	1.39
Low complexity		9,452	1,843,813	0.33
Total base masked		131,988	24,335,696	17.37

Table S7. Genome features of the *M. cingulum, N. vitripennis and A. mellifera*

Genomic features	M. cingulum (version 1.0)	N. vitripennis (version 1.0)	A. mellifera（version 3.2）
genome size (Mb)	132	295	227
CEGMA genes (%)	99.2	97.6	98.8
Contig N50 (bp)	64,884	18,500	42,850
Scaffold N50(bp)	192,445	709,000	986,335
GC content (%)	35.7	40.6	32.8
Repeat rate (%)	24.9	42.1	13.6
Coding gene number	11,993	18,822	15,314
Number of genes with Go terms	7,254	8,778	8,162
Number of genes with InterPro domains	9,094	17,079	9,249
Number of miRNA	111	953	314
Number of tRNA	144	756	271
Number of snoRNA	16	71	53
Number of rRNA	148	47	38

Table S8. Gene features of *M. cingulum*, *N. vitripennis* and *A. mellifera*

Species	Exon Number	Exon Length	Gene Length	Intron Length	CDS Length
M. cingulum	5.13	299.61	3127.88	385.13	1536.40
N. vitripennis	5.46	284.13	7281.90	1285.14	1289.52
A. mellifera	5.53	320.29	7615.47	1291.25	1260.73

Table S9. The insects with OGSs in InsectBase

Species name	Genome size (Mb)	Gene number	Protein number
Acromyrmex echinatior	289	17,277	17,277
Acyrthosiphon pisum	525	36,194	36,938
Aedes aegypti	1,434	18,800	17,142
Anopheles coluzzii	219	14,702	14,702
Anopheles darlingi	133	10,947	10,456
Anopheles gambiae	265	15,423	14,666
Anopheles sinensis	215	19,352	19,351
Anopheles stephensi	219	13,787	13,250
Apis dorsata	223	20,601	18,144
Apis florea	224	19,333	17,663
Apis mellifera	227	15,313	15,313
Athalia rosae	159	20,151	19,484
Atta cephalotes	309	18,092	18,092
Bactrocera cucurbitae	363	21,640	20,753
Bactrocera dorsalis	402	18,231	17,744
Bombus impatiens	242	21,775	20,895
Bombus terrestris	241	21,558	20,320
Bombyx mori	467	14,622	14,622
Camponotus floridanus	228	17,063	17,063
Cerapachys biroi	206	26,315	23,802
Ceratitis capitata	469	23,710	23,087
Ceratosolen solmsi	268	13,200	12,833
Chilo suppressalis	362	10,221	10,221
Culex quinquefasciatus	562	19,389	19,018
Danaus plexippus	238	15,129	15,129
Dendroctonus ponderosae	246	13,456	13,459
Diaphorina citri	492	21,985	20,995
Drosophila ananassae	225	15,588	15,070
Drosophila erecta	148	15,615	15,048
Drosophila grimshawi	196	15,415	14,986
Drosophila melanogaster	139	17,717	30,439
Drosophila mojavensis	188	14,990	14,595
Drosophila persimilis	184	17,351	16,878
Drosophila pseudoobscura	149	17,382	16,857
Drosophila sechellia	163	17,049	16,483
Drosophila simulans	135	15,912	15,427
Drosophila virilis	201	15,343	14,491
Drosophila willistoni	230	16,008	15,513
Drosophila yakuba	161	16,515	16,094

Fopius arisanus	149	20,056	18,906
Harpegnathos saltator	288	18,563	18,563
Heliconius melpomene	266	15,201	12,828
Linepithema humile	213	16,115	16,115
Manduca sexta	367	27,529	27,402
Mayetiola destructor	180	22,630	22,024
Megachile rotundata	264	28,682	26,024
Megaselia scalaris	489	11,638	11,462
Microplitis demolitor	243	18,954	18,169
Musca domestica	728	20,165	19,549
Nasonia vitripennis	287	18,940	18,821
Nilaparvata lugens	1,324	36,723	36,723
Pediculus humanus	108	11,664	10,775
Plutella xylostella	383	18,072	18,072
Pogonomyrmex barbatus	228	17,737	17,188
Rhodnius prolixus	683	15,642	15,440
Solenopsis invicta	343	16,521	16,521
Tribolium castaneum	222	16,542	16,525
Vollenhovia emeryi	280	27,195	26,071
Wasmannia auropunctata	323	26,070	24,085
Zootermopsis nevadensis	472	14,610	14,610

Table S10. Hemomucin genes in eight wasps

Order	Species	SRA	Length	O-glycosylation positions
Braconidae	Microplitis bicoloratus	9	443	7
Braconidae	Microplitis demolitor	4	444	9
Braconidae	Macrocentrus cingulum		579	56
Braconidae	Cotesia vestalis	12	439	3
Braconidae	Cotesia rubecula	12	439	4
Braconidae	Cotesia glomerata	30	439	5
Ichneumonidae	Venturia canescens	1	616	65
Ichneumonidae	Diadromus collaris	2	436	5

Table S11. The different gene expression of Embryo and Pseudogerm transcriptomes in KEGG pathway

Pathway map 1-categories	Pathway map 2-categories	Embryo upregulate	Pseudogerm upregulate
Metabolism	Global and overview maps	2	1
	Carbohydrate metabolism	6	12
	Energy metabolism	1	2
	Lipid metabolism	3	4
	Nucleotide metabolism	5	1
	Amino acid metabolism	9	4
	Metabolism of other amino acids	4	2
	Glycan biosynthesis and metabolism	5	4
	Metabolism of cofactors and vitamins	0	1
	Metabolism of terpenoids and polyketides	0	3
	Biosynthesis of other secondary metabolites	0	0
	Xenobiotics biodegradation and metabolism	0	1
Genetic Information Processing	Transcription	0	0
	Translation	2	1
	Folding, sorting and degradation	2	3
	Replication and repair	3	0

Environmental Information Processing	Membrane transport	2	1
	Signal transduction	39	28
	Signaling molecules and interaction	4	1
Cellular Processes	Transport and catabolism	7	7
	Cell motility	1	0
	Cell growth and death	8	1
	Cellular community	15	5
Organismal Systems	Immune system	9	5
	Endocrine system	14	14
	Circulatory system	4	1
	Digestive system	5	6
	Excretory system	1	2
	Nervous system	3	7
	Sensory system	0	4
	Development	3	1
	Environmental adaptation	0	3
Human Diseases	Cancers: Overview	14	9
	Cancers: Specific types	15	6

Immune diseases	1	2
Neurodegenerative diseases	1	4
Substance dependence	0	1
Cardiovascular diseases	3	0
Endocrine and metabolic diseases	2	3
Infectious diseases: Bacterial	9	5
Infectious diseases: Viral	8	2
Infectious diseases: Parasitic	6	0

Table S12. The differently expressed miRNAs in embryo and mixed embryo transcriptomes

miRNA	Normal embryo	Mixed embryo	log2FoldChange	pvalue
mci-miR-14b	20.21998745	1991.692631	4.924602884	0.015168939
mci-miR-9a-5p	1.263749216	42.72999685	3.446504164	0.111932706
mci-miR-9a-3p	1.263749216	34.02573823	3.189647009	0.144792447
mci-miR-1a	0	14.24333228	3.218293882	0.174454125
mci-miR-6038	10.10999373	0	-2.944791637	0.217081046
mci-bantam-5p	7.582495294	0	-2.553140638	0.285047192
mci-miR-210a-3p	7.582495294	0	-2.553140638	0.285047192
mci-miR-iab-4-3p	0	7.912962379	2.472083197	0.300695842
mci-miR-1175	1.263749216	15.03462852	2.23409632	0.327254046
mci-miR-932	6.318746078	0	-2.308083435	0.332374298
mci-miR-2491c	41.70372412	7.912962379	-1.828253143	0.366669062
mci-miR-3770	17.69248902	69.63406894	1.563998291	0.419362271
mci-miR-6065	115.0011786	317.3097914	1.207760725	0.505654641
mci-miR-2779a	0	3.165184952	1.398284856	0.537206421
mci-miR-993-3p	0	3.165184952	1.398284856	0.537206421
mci-miR-iab-8-3p	0	3.165184952	1.398284856	0.537206421
mci-bantam-3p	2.527498431	0	-1.36733031	0.541944226
mci-miR-210b	2.527498431	0	-1.36733031	0.541944226
mci-miR-281-5p	5.054996863	0.791296238	-1.312692382	0.582609892
mci-miR-4945	11.37374294	3.165184952	-1.219072159	0.588920427
mci-miR-996	0	2.373888714	1.195588272	0.593695913
mci-miR-2779f	1.263749216	0	-1.081479192	0.62619967
mci-miR-3477-3p	1.263749216	0	-1.081479192	0.62619967

mci-miR-971	1.263749216	0	-1.081479192	0.62619967
mci-miR-2491a	34.12122882	15.03462852	-0.918543525	0.644005878
mci-miR-252-3p	0	1.582592476	0.970549781	0.664401652
mci-miR-263a	0	1.582592476	0.970549781	0.664401652
mci-miR-71a	2.527498431	7.912962379	1.005734728	0.665065447
mci-miR-2491b	69.50620686	34.81703447	-0.805577886	0.668644215
mci-miR-6067	61.92371157	30.86055328	-0.808089568	0.669759722
mci-miR-33	5.054996863	1.582592476	-0.899790707	0.705393978
mci-miR-276	0	0.791296238	0.596960254	0.788636567
mci-miR-277-5p	0	0.791296238	0.596960254	0.788636567
mci-miR-305	0	0.791296238	0.596960254	0.788636567
mci-miR-307-5p	0	0.791296238	0.596960254	0.788636567
mci-miR-87	0	0.791296238	0.596960254	0.788636567
mci-miR-iab-4-5p	0	0.791296238	0.596960254	0.788636567
mci-miR-6001	8.846244509	4.747777427	-0.594455326	0.792409835
mci-miR-6006	5.054996863	8.704258617	0.519521645	0.817976599
mci-miR-2491d	5.054996863	7.912962379	0.423834485	0.851835053
mci-miR-308	18.95623823	15.03462852	-0.253592447	0.901323242
mci-miR-278	1.263749216	0.791296238	-0.22379999	0.92081334
mci-miR-277-3p	2.527498431	3.165184952	0.16854129	0.943714403
mci-miR-34	1.263749216	1.582592476	0.123433982	0.957577503
mci-miR-281-3p	5.054996863	5.539073665	0.082910259	0.971353955
mci-miR-184	5.054996863	4.747777427	-0.0557747	0.980846862
mci-miR-12	2.527498431	2.373888714	-0.044437896	0.985157543
mci-miR-282	32.85747961	33.23444199	0.01309561	0.994576002
mci-miR-375	24.0112351	23.73888714	-0.012839458	0.994822876

Table S13. Comparison of gene numbers for Chemoreception in *A.mellifera, C. floridanum, C. solmsi, M. cingulum, N. vitripennis* and *S. invicta*

Organisms	IR	SNMP	CSP	OR	OBP	GR
A. mellifera	24	10	9	142	21	18
C. floridanum	33	20	9	80	40	24
C. solmsi	29	11	8	49	10	6
M. cingulum	25	9	3	89	7	14
N. vitripennis	32	12	10	131	90	31
S. invicta	24	10	15	125	16	28

Table S14. Comparison of gene numbers for Gene families associated with insecticide resistance and detoxification in *D. melanogaster*, *A. mellifera*, *C. floridanum*, *C. solmsi*, *M. cingulum*, *N. vitripennis* and *S. invicta*

Organisms	P450	GST	ABC
D. melanogaster	85	35	59
A. mellifera	56	14	64
C. floridanum	108	22	99
C. solmsi	46	17	78
M. cingulum	43	15	68
N. vitripennis	96	26	62
S. invicta	133	12	69

Table S15. Comparison of gene numbers of insect immune in *A. mellifera*, *C. floridanum*, *C. solmsi*, *M. cingulum*, *N. vitripennis* and *S. invicta*

Genes	*A. mellifera*	*C. floridanum*	*C. solmsi*	*D. melanogaster*	*M. cingulum*	*N. vitripennis*	*S. invicta*
CASPs	5	6	9	7	2	8	7
CASPAs	1	1	1	1	2	1	1
CATs	3	1	1	2	0	1	1
CTLs	13	22	25	41	19	32	11
FREPs	2	1	1	14	2	1	2
GALEs	3	5	3	6	3	3	2
IAPs	5	12	5	4	4	17	6
LYSs	2	3	1	12	1	1	1
MLs	4	2	3	8	4	7	5
PRRs	6	11	6	17	6	12	5
GNBPs	2	1	0	6	2	2	2
PGRPs	4	10	6	11	4	10	3
PPOs	5	5	8	10	3	10	4
RELs	4	7	7	4	3	6	2
SPZs	7	13	9	6	5	9	4
TEPs	4	5	3	6	3	3	3
TOLLs	8	9	6	9	13	10	4

CASPs: caspases, CASPAs: caspase Activators, CATs:Catalases, CTLs: C-type lectins, FREPs: Fibrinogen-Related Proteins, GALEs: Galactoside-Binding Lectins, IAPs: Inhibitors of Apoptosis, LYSs: Lysozymes, MLs: MD2-Like Receptors, PPOs: prophenoloxidases, PRRs: Pattern Recognition Receptors, GNBPs: Gram-negative binding proteins, PGRPs: peptidoglycan recognition proteins, RELs: Relish-like Proteins, SODs: Superoxide Dismutatses, Spzs: Spaetzle-like Proteins, TEPs: thioester-containing protein, TOLLs: Toll-Receptors

Table S16. The PCR primer for target genes of mci-miR-14b

Genes	Primer
MBP-1	TTTACGGTTCGGCTATCACTTT
	CGGCATCGCTAGATCCTCA
KMT2E	TTGAAATCGTGCGTGGTCTT
	GAGGGCTCATCGTAGGTGGTA
RUNT	CCAGTCCAACACCCACGAA
	TGGCTTACACTTATGCCTCCTAT
